# Side-chain Engineering of Benzo[1,2-b:4,5-b’]dithiophene Core-structured Small Molecules for High-Performance Organic Solar Cells

**DOI:** 10.1038/srep25355

**Published:** 2016-05-03

**Authors:** Xinxing Yin, Qiaoshi An, Jiangsheng Yu, Fengning Guo, Yongliang Geng, Linyi Bian, Zhongsheng Xu, Baojing Zhou, Linghai Xie, Fujun Zhang, Weihua Tang

**Affiliations:** 1 Key Laboratory of Soft Chemistry and Functional Materials (Ministry of Education of China), Nanjing University of Science and Technology, Nanjing, 210094, China; 2 Key Laboratory of Luminescence and Optical Information (Ministry of Education of China), Beijing Jiaotong University, Beijing, 100044, China; 3 Key Laboratory for Organic Electronics and Information Displays (KLOEID) and Institute of Advanced Materials (IAM), Jiangsu National Synergetic Innovation Center for Advanced Materials (SICAM), Nanjing University of Posts and Telecommunications, 9 Wenyuan Road, Nanjing 210046, China

## Abstract

Three novel small molecules have been developed by side-chain engineering on benzo[1,2-b:4,5-b’]dithiophene (BDT) core. The typical acceptor-donor-acceptor (A-D-A) structure is adopted with 4,8-functionalized BDT moieties as core, dioctylterthiophene as π bridge and 3-ethylrhodanine as electron-withdrawing end group. Side-chain engineering on BDT core exhibits small but measurable effect on the optoelectronic properties of small molecules. Theoretical simulation and X-ray diffraction study reveal the subtle tuning of interchain distance between conjugated backbones has large effect on the charge transport and thus the photovoltaic performance of these molecules. Bulk-heterojunction solar cells fabricated with a configuration of ITO/PEDOT:PSS/SM:PC_71_BM/PFN/Al exhibit a highest power conversion efficiency (PCE) of 6.99% after solvent vapor annealing.

Organic solar cells (OSCs) have attracted great interest due to their potential in cost-effectively fabricating lightweight and flexible panels in large area via solution-processing techniques^1^,^2^. Donor materials including both conjugated polymers and small molecules (SMs) have been extensively developed for the bulk heterojunction (BHJ) structured OSCs^3^,^4^. Great progress has been made in both materials development and device optimization, witnessed by the dramatic improvement in device power conversion efficiencies (PCEs) from less than 2% before 2008 to over 10% in recent three years^5^,^6^,^7^,^8^,^9^. In comparison to polymer donors, conjugated small molecules boast well-defined structure to eliminate the structural variation in terms of regioregularities, molecular weight and polydispersity. Moreover, small molecules can be more easily designed to meet the criteria of good donors for BHJ solar cells, such as strong absorption, suitable energy levels, high hole mobility and solubility^10^,^11^,^12^. One of the challenges in developing high-efficiency small molecules OSCs is the generation of nanoscale phase-segregated donor-acceptor (D-A) heterojuction in the active layer. The ideal morphology featuring bicontinuous networking with phase separation in the scale commensurating with the exciton diffusion length^13^. In device optimization, the morpholohical tuning of vertical segregation and domain conectivity as well as horizontal phase separation in BHJ are found to be critical^14^. Devices engineering such as appropriate solvent screening, additives, solvent vapor annealing, thermal annealing and fullerene choice has demonstrated great potential in enhancing OSC performance^6^,^8^,^10^,^15^,^16^,^17^,^18^,^19^.

Benefited from the D-A alternating polymers design to achieve a good balance of suitable bandgap and and charge carrier mobility^1^,^2^,^5^, small conjugated molecules with mutiple D-A chromophores have been successfully designed for high-efficiency OSCs^3^,^4^,^8^,^9^,^10^,^11^,^12^. Chen and his coworkers have explored a large library of solution-processed A-D-A type SMs, with central D units varied from oligothiophene to benzo[1,2-b:4,5-b’]dithiophene (BDT) and dithienosilole (DTS), while teminal A units like alkyl cyanoacetate and 3-ethylrhodanine were adopted^3^,^9^,^18^,^19^,^20^,^21^. Bazan *et al.*^16^,^22^ have recently explored D1–A–D2–A–D1 structured SMs, featuring DTS or silaindacenodithiophene as D2 core while benzothiadiazole (BT) or its fluorinated derivatives as A unit and oligothiophenes as terminal D1. BDT was chosen as the core unit by taking advantage of its large and rigid coplanar π-conjunction system, which can lead to efficient π-π stacking for good charge transport^3^,^4^,^5^,^6^,^8^,^9^,^10^. On the other hand, by introducing conjugated side chains to the 4- and 8- position of BDT, two dimensional BDT (2-D BDT) was obtained to achieve lower-lying HOMO (highest occupied molecular orbital) level thus higher *V*_*oc*_, extended absorption thus higher *J*_*sc*_ and better charge transport^23^,^24^. The impact of 4,8-difuntionalities on BDT core on the resultant D-A polymers donors for OSC application has been extensively investigated^24^,^25^. By varying the side chain from alkyl to alkylthio or alkylthienyl, the BDT based polymers may ehibit substantially improved hole mobility and subtle change in energy levels^25^.

To tackle the challenge in manipulating bandgap and hole mobility of BDT donating cores, a versatile synthetic platform has been developed for the 4,8-disubstitution of BDT in our group, where the charge transporting BDTs exhibit the energy levels fine-tuned with functionalization^26^. For BDT based D-A polymers, the structure modification on BDT has great effect on their energy levels, hole mobility and molecular packing of polymer backbones^24^,^25^. For SM-OSCs, it is very difficult to achieve appropriate phase separation in active layers for SM donors with short conjugated backbone. We are intrigued whether the substitution on BDT core can improve the charge transportation of D-A structured SMs by promoting better molecular packing and phase segregation when blending with fullerene in OSCs.

We report herein three novel A-D-A structured SMs (ca. **DR3TBDTOC12, DR3TBDTTC12** and **DR3TBDTTSC8**) featuring side-chain engineered BDT core and electron-withdrawing 3-ethylrhodanine end group (as shown in **Scheme 1**). Among them, **DR3TBDTTC12** and **DR3TBDTTSC8** have a corresponding alkylthienyl- and alkylthiothienyl-substituted BDT unit to construct 2-D BDT core structures. The impact of 2-D BDT cores on the optoelectronic properties of the SMs was investigated with experimental studies and molecular simulation. The photovoltaic performance of these SMs was further evaluated in BHJ solar cells. The SM-OSCs fabricated by blending alkylthienyl- or alkylthiothienyl-substituted 2-D BDT cored SMs with [6,^6^]-phenyl-C_71_-butyric acid methyl ester (PC_71_BM) exhibited much higher PCEs than the corresponding OSCs with alkyl-BDT cored SM. A highest PCE of 6.99% was achieved, with a *J*_*sc*_ of 11.69 mA cm^−2^, a *V*_*oc*_ of 0.90 V and a fill factor (FF) of 66.48%.

## Experiment Section

### Characterization

^1^H NMR and ^13^C NMR spectra were recorded on Bruker AVANCE 500-MHz spectrometer with tetramethylsilane (TMS) as internal standard. Matrix assisted laser desorption/ionization time-of-flight (MALDI-TOF) mass spectra were obtained on a Bruker Autoflex TOF/TOF spectrometer. UV-vis absorption spectra were recorded on a UV-Vis instrument Evolution 220 (Thermo Fisher). XRD analysis was performed on a Bruker D8 Advance X-Ray Diffractometer in air. CV measurement was conducted on an electrochemical workstation (CHI660D Chenhua Shanghai) with Pt plate as working electrode, Pt slice as counter electrode, and Ag/AgCl electrode as reference electrode in a Bu_4_NPF_6_ (0.1 M) acetonitrile solution at a scan rate of 50 mV s^−1^. Ferrocene/ferrocenium (Fc/Fc^+^) was used as the internal standard (the energy level of Fc/Fc^+^ is −4.8 eV under vacuum)^20^, and the formal potential of Fc/Fc^+^ was measured as 0.49 V vs. Ag/AgCl electrode. TGA analysis was conducted under nitrogen atmosphere at a heating rate of 20 °C min^−1^ from 50 °C to 800 °C. The instrument type was TGA/SDTA851E (Mettler Toledo). The morphology of blend films was investigated by Atomic Force Microscope (Bruker Dimension Icon) in tapping mode.

### Device Fabrication and Testing

The patterned ITO glass substrates (sheet resistance 15 Ω/□) were cleaned consecutively in ultrasonic baths containing acetone, detergent, de-ionized water and ethanol, respectively. The cleaned ITO substrates were blow-dried using high pure nitrogen gas before treated by UV-ozone for 10 min to further increase its work function. Onto the freshly cleaned ITO substrates was spin-coated the solution of PEDOT:PSS (purchased from H.C. Starck co. Ltd.) at 5000 round per minute (RPM) for 40 s to make the interfacial layers. The PEDOT:PSS coated ITO glass substrates were then annealed at 150 °C for 10 min in air environment. The electron donor materials SM and electron acceptor materials PC_71_BM were dissolved in chloroform to generate 20 mg ml^−1^ blend solutions. The weight ratio of SM:PC_71_BM was kept constant as 1:1 (w:w). The blend solutions were spin-coated on PEDOT:PSS films at 800 RPM for 30 s in a high purity nitrogen-filled glove box to fabricate the active layers. The thickness of the active layers is around 90 nm, which is measured by Ambios Technology XP-2 stylus Profiler. The polyelectrolyte poly[9,9-bis(3′-(N,N-dimethylamino)-propyl-2,7-fluorene)-alt-2,7-(9,9-dioctylfluorene)] (PFN) was then dissolved in methanol to prepare 0.2 mg ml^−1^ solution with addition of 0.25% (volume) acetic acid, and then the PFN solutions were spin-coated on the top of active layers at 3000 RPM for 30 s. The cathode of Al (100 nm) film was deposited on the PFN film by thermal evaporation under 10^−4^ Pa and the thickness was monitored by a quartz crystal microbalance. The active area is about 3.8 mm^2^, which is defined by the vertical overlap of ITO anode and Al cathode.

The *J–V* curves of all solar cells were measured in air environment using a Keithley 2400 source meter under AM 1.5G (100 mW cm^−2^) irradiation provided by an ABET Sun 2000 solar simulator. The EQE spectra of solar cells were measured by a Zolix Solar Cell Scan 100.

### Synthesis

**DR3TBDTOC12.** Compound **10** (200 mg, 0.12 mmol) was dissolved in dry CHCl_3_ (50 mL), three drops of piperidine and 3-ethylrhodanine (207.18 mg, 1.2 mmol) were added. The resulting solution was heated to reflux and stirred for 12 h under N_2_. After cooling to room temperature, the mixture was then extracted with CH_2_Cl_2_, washed with water and brine, and dried over Mg_2_SO_4_. After removal of solvent, it was purified by column chromatography using CHCl_3_ as eluent, the crude product was recrystallized from acetone to afford **DR3TBDTOC12** (170 mg, 72%) as a dark red solid. ^1^H NMR (500 MHz, CDCl_3_, δ/ppm): 7.74 (s, 2 H), 7.39 (s, 2 H), 7.20 (s, 4 H), 7.11 (s, 4 H), 4.28 (s, 4 H), 4.17 (q, *J* = 7.0 Hz, 4 H), 2.79 (s, 8 H), 2.03–1.84 (m, 4 H), 1.70 (dd, *J* = 14.9, 7.4 Hz, 8 H), 1.53–1.07 (m, 82 H), 0.97–0.71 (m, 18 H). ^13^C NMR (126 MHz, CDCl_3_, δ/ppm): 192.01, 167.27, 143.87, 141.02, 139.46, 137.51, 137.27, 136.13, 135.52, 135.19, 134.78, 132.53, 130.39, 129.30, 128.32, 127.19, 126.19, 124.83, 120.56, 116.13, 73.88, 39.91, 31.94, 30.60, 30.48, 30.26, 29.73, 29.62, 29.53, 29.42, 26.11, 22.71, 14.14, 12.31. MALDI-TOF MS: calcd. for C_102_H_140_N_2_O_4_S_12_ *m*/*z* = 1841.75; found 1841.93.

**DR3TBDTTC12.** Following the same procedure above, the condensation between **11** (200 mg, 0.12 mmol) and 3-ethylrhodanine (190.96 mg, 1.2 mmol) afforded **DR3TBDTTC12** (158 mg, 67%) as a dark brown solid. ^1^H NMR (500 MHz, CDCl_3_, δ/ppm): 7.76 (s, 2 H), 7.60 (s, 2 H), 7.31 (d, *J* = 2.9 Hz, 2 H), 7.21 (d, *J* = 5.9 Hz, 4 H), 7.11 (s, 4 H), 6.97 (s, 2 H), 4.18 (q, *J* = 7.1 Hz, 4 H), 2.97 (t, *J* = 7.6 Hz, 4 H), 2.86–2.64 (m, 8 H), 1.91–1.78 (m, 4 H), 1.67 (d, *J* = 7.0 Hz, 6 H), 1.56–1.44 (m, 8 H), 1.32 (dd, *J* = 36.3, 29.3 Hz, 76 H), 0.98–0.79 (m, 18 H). ^13^C NMR (126 MHz, CDCl_3_, δ/ppm): 192.03, 167.28, 147.44, 141.04, 140.96, 139.47, 138.71, 137.60, 137.37, 137.26, 136.60, 135.59, 135.19, 134.74, 130.45, 128.39, 127.97, 127.23, 126.19, 124.83, 124.40, 123.34, 120.58, 119.14, 39.91, 31.93, 31.62, 30.44, 30.38, 30.26, 29.71, 29.58, 29.46, 29.32, 22.70, 14.13, 12.30. MALDI-TOF MS: calcd. for C_110_H_144_N_2_O_4_S_14_ *m/z* = 1973.74; found 1973.91.

**DR3TBDTTSC8.** Following the same procedure above, the condensation between **12** (200 mg, 0.12 mmol) and 3-ethylrhodanine (195.56 mg, 1.2 mmol) afforded **DR3TBDTTSC8** (150 mg, 64%) as a black solid. ^1^H NMR (500 MHz, CDCl_3_, δ/ppm): 7.66 (d, *J* = 6.2 Hz, 2 H), 7.43 (d, *J* = 11.1 Hz, 2 H), 7.33 (d, *J* = 13.5 Hz, 2 H), 7.28 (s, 2 H), 7.12 (s, 4 H), 7.00 (dd, *J* = 26.2, 15.8 Hz, 4 H), 4.14 (dd, *J* = 14.0, 6.9 Hz, 4 H), 3.02 (t, *J* = 6.9 Hz, 4 H), 2.83–2.60 (m, 8 H), 1.97–1.74 (m, 4 H), 1.65 (s, 8 H), 1.52 (dt, *J* = 14.9, 7.4 Hz, 6 H), 1.32 (dt, *J* = 14.4, 7.2 Hz, 60 H), 0.96–0.78 (m, 18 H). ^13^C NMR (126 MHz, CDCl_3_, δ/ppm): 191.91, 167.18, 141.86, 140.82, 139.49, 138.43, 137.21, 135.02, 134.76, 132.74, 128.54, 127.03, 126.00, 124.68, 122.57, 120.47, 118.50, 39.88, 38.91, 31.91, 31.83, 30.41, 30.18, 29.81, 29.60, 29.50, 29.35, 29.25, 28.63, 22.70, 14.14, 12.30. MALDI-TOF MS: calcd. for C_102_H_128_N_2_O_2_S_16_ *m/z* = 1925.55; found 1973.67.

## Results and Discussion

### Synthesis

**Figure 1:**
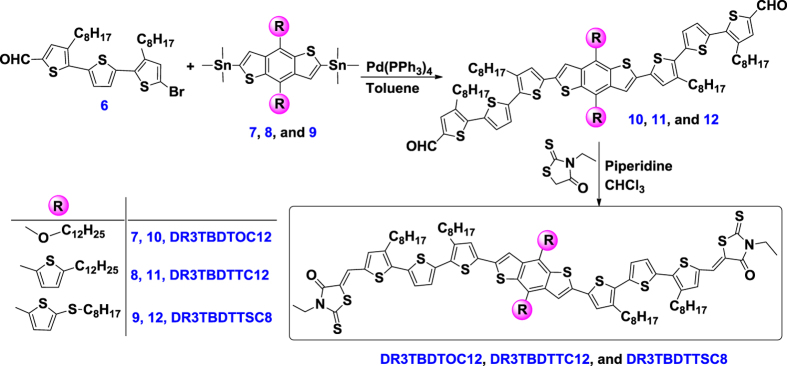
Synthetic routes of DR3TBDTOC12, DR3TBDTTC12 and DT3TBDTTSC8.

Different side-chain engineered BDT-core based SMs were prepared using a two-step synthetic pathway as shown in Fig. 1. For the consideration of solubility and hole transport, dioctylterthiophene π-conjugated bridge **6** as reported in literature^8^,^27^, was adopted to insert between BDT core and rhodanine terminal group. Side-chain engineered BDTs **7–9** were readily obtained according to our previous report^26^,^28^. The detailed synthesis of various intermediates for compounds **6–9** was provided in Supporting Information (SI). With **6** and BDT core (**7–9**) at hand, the typical Stille cross-coupling reaction afforded the π-D-π structured intermediate compounds **10–12** with a yield of 49**–**60%. Further Knoevenagel condensation between rhodanine and **10–12** was further conducted to afford the title A-D-A SMs with good yields (64**–**72%). The structure of all SMs was confirmed with NMR and MALDI-TOF mass spectra.

**Figure 2:**
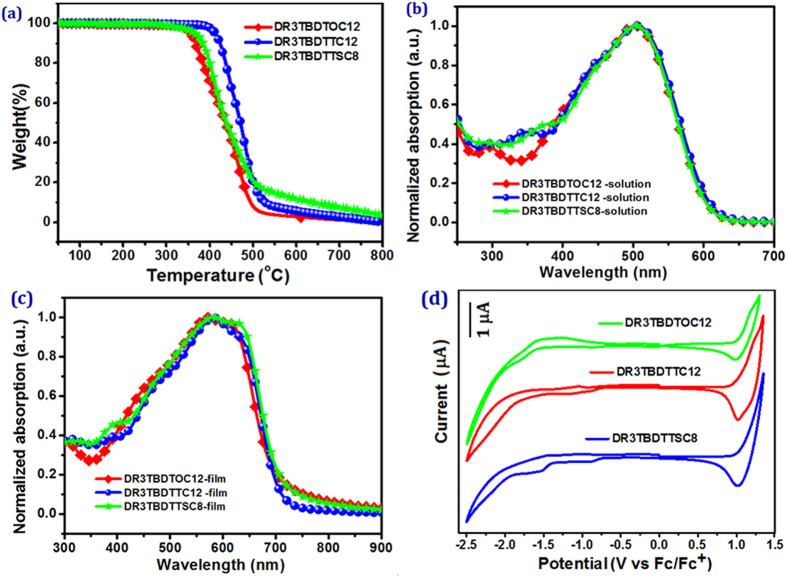
(**a**) TGA plots of DR3TBDTOC12, DR3TBDTTC12 and DT3TBDTTSC8; (**b**) UV-vis absorption spectra of three SMs in chloroform solutions; (**c**) UV-vis absorption spectra of three SMs in solid films; (**d**) cyclic voltammograms of three SMs in a acetonitrile solution of 0.1 mol L^−1^ Bu_4_NPF_6_ with a scan rate of 50 mV s^−1^.

All SMs show good solubility in common organic solvents like chloroform, chlorobenzene and dichlorobenzene. Thermogravimetric analysis (TGA) revealed the good thermal stability for these SMs. The decomposition temperature (5% weight loss) of 354, 366 and 410 °C in nitrogen was observed for **DR3TBDTOC12**, **DR3TBDTTSC8** and **DR3TBDTTC12**, respectively (Fig. 2a). This is beneficial for the device fabrication for their optoelectronic application^29^,^30^.

### Optical and Electrochemical Properties

The UV-Vis absorption spectra of SMs in dilute chloroform solutions and thin films are shown in Fig. 2c,d. The characteristic absorption data, including absorption maxima in solutions and films, as well as absorption onsets and bandgaps for films are summarized in Table 1.

Though different side-chains were employed for the substitution of BDT core, all SMs exhibit quite similar absorption profile in solutions and films. In solutions, SMs show an absorption range of 300~630 nm with an absorption peak at 502**–**504 nm, where 2-D BDT based **DR3TBDTTSC8** and **DR3TBDTTC12** exhibited only 2~3 nm red-shifted absorption maxima than alkoxyl BDT based **DR3TBDTOC12**. The absorption bands of SMs are extended to 730 nm for their films. This bathochromic shift indicates the formation of effective π-π packing between the molecule backbones in solid film^31^. This red shift for 2-D BDT based SMs films increased to 11~12 nm in comparision to 1-D **DR3TBDTOC12**. It is worth to note that alkylthiothienyl based **DR3TBDTTSC8** presents a ~9 nm and 13 nm broader absorption band than alkylthienyl-based **DR3TBDTTC12** and alkoxy-substituted **DR3TBDTOC12**. Besides a red-shifted main peak, all SMs films exhibit a vibronic shoulder at 627, 628 and 612 nm for **DR3TBDTTC12**, **DR3TBDTTSC8** and **DR3TBDTOC12**, respectively. From the absorption onset values of SMs’ films, the optical bandgap (*Eg*^*opt*^)^31^,^32^ was estimated as 1.76, 1.75 and 1.73 eV for **DR3TBDTOC12**, **DR3TBDTTC12** and **DR3TBDTTSC8**, respectively. It is noted that the side-chain engineering exerts negligible impact on the bandgap of SMs. But 2-D BDT cored SMs showed red-shifted absorption peaks in comparison to 1D-BDT counterpart, which may be beneficial to its light-harvesting properties in OSCs.

To check the differences in energy levels with the change of side chains, the HOMO energy levels and the LUMO (lowest unoccupied molecular orbital) levels of **DR3TBDTOC12**, **DR3TBDTTC12**, **DR3TBDTTSC8** were determined by cyclic voltammetry (CV). As shown in Fig. 2b, clear oxidation and reduction peaks were observed for SMs. From the onset potentials for oxidation (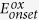)^31^, the HOMO energy level was determined to be −5.32, −5.34, and −5.38 eV for **DR3TBDTOC12**, **DR3TBDTTC12** and **DR3TBDTTSC8**, respectively. The corresponding LUMO energy level was determined from the onset potentials for reduction (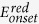) as −3.59, −3.63, and −3.65 eV, respectively. Thus, the electrochemical bandgaps (*Eg*^*cv*^) of **DR3TBDTOC12**, **DR3TBDTTC12** and **DR3TBDTTSC8** was thus calculated to be 1.73, 1.71 and 1.73 eV. The *Eg*^*cv*^ values of three SMs agree well with the optical bandgap. The side chain replacement from alkoxy to alkylthienyl and alkylthiothineyl group on BDT core, the HOMO level of resultant SMs was reduced from −5.32 to −5.34 and −5.38 eV. This result showed that the extension of conjugation length using 2-D BDT design lead to deeper HOMO level, and the insertion of sulfur atom in side chain would further lower down the HOMO of SM. The decreased HOMO level is beneficial for the *V*_*oc*_ of OSCs, thus leading to better device performance^2^,^24^.

### X-Ray Diffraction (XRD) Characterization and Theoretical Calculation

**Figure 3:**
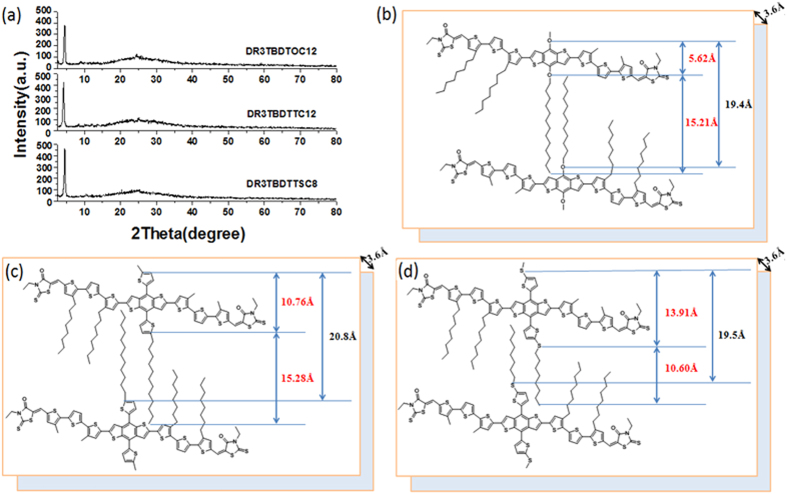
(**a**) XRD patterns of SMs powder and visualized image of molecular packing by side-chains and π-stacking distance of (**b**) DR3TBDTOC12, (**c**) DR3TBDTTC12 and (**d**) DR3TBDTTSC8. The distance in black are XRD results and those values in red are estimated by the DFT calculation.

The crystallization behavior of the as-synthesized SMs in solid state was investigated using XRD technology on powder sample of SMs (Fig. 3a). Sharp peak corresponding to 2θ = 4.5°, 4.2° and 4.5° is observed for **DR3TBDTOC12**, **DR3TBDTTC12** and **DR3TBDTTSC8**, respectively, indicating an ordered molecular structure^33^,^34^,^35^. The distance between SMs conjugated backbone separated by side chains can thus be calculated to be 19.4, 20.7, and 19.5 Å, respectively^33^,^36^. All of them also showed a weak diffraction at 2θ = 24.6°, indicating the π-stacking distance between planar backbones is about 3.6 Å^37^. The visualized images of molecular packing for three SMs (Fig. 3b–d) are shown with interchain and π-stacking distance from XRD analysis and DFT calculation^37^. **DR3TBDTOC12** with two dodecoxy chains on BDT core showed that the interchain distance observed by XRD is almost equivalent to the length of the dodecoxy estimted by the DFT calculation. For 2-D BDT cored SMs (**DR3TBDTTC12** and **DR3TBDTTSC8**), however, the interchain distances observed by XRD are rather shorter than the the length of alkylthineyl and alkylthiothienyl side chains at BDT estimated by DFT calculation, indicating the more densely packing occuring in 2-D BDT cored SMs to reduce the space adequately.

**Figure 4:**
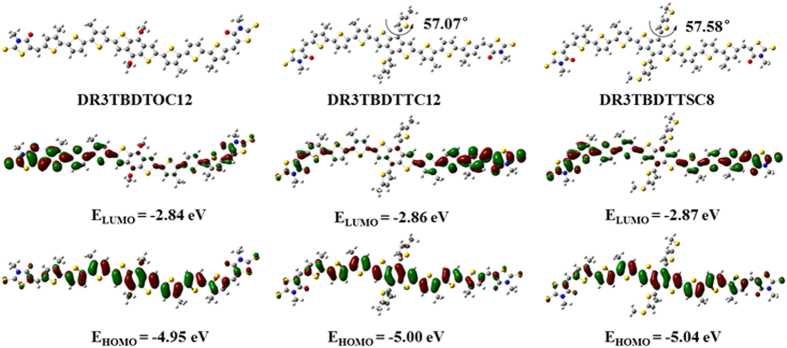
Optimized molecular geometries and frontier molecular orbitals of SMs.

The electronic structures and geometries of SMs was also investigated with DFT calculation using Gaussian program at the B3LYP/6-31G (d,p) level^27^,^38^. All alkyl side chains were replaced with methyl groups to reduce the computational cost. Three SMs showed good planarity in conjugated backbone (Fig. 4). The introduction of alkylthienyl and alkylthiothienyl side groups, leads to a torsion angle of 57.07° and 57.58° between side-chain and mainchain. The torsion of side-chains around BDT core in **DR3TBDTTC12** and **DR3TBDTTSC8** may facilitate their higher dense packing along interchains, as revealed by XRD study. The electron density of LUMO mainly concentrates on terthiophene and rhodanine moieties, while HOMO mainly localizes on BDT and terthiophene portions. The HOMO/LUMO energy levels of **DR3TBDTOC12**, **DR3TBDTTC12** and **DR3TBDTTSC8** were estimated at showed a HOMO energy level of −4.95/−2.84, −5.00/−2.86 and −5.04/−2.87 eV, respectively. The bandgaps of SMs were accordingly calculated as 2.11, 2.14 and 2.17 eV. Obviously, the trends in bandgap and energy levels (both HOMO and LUMO) are consistent with those observed in UV-vis absorption and CV studies.

### Photovoltaic Properties

**Figure 5:**
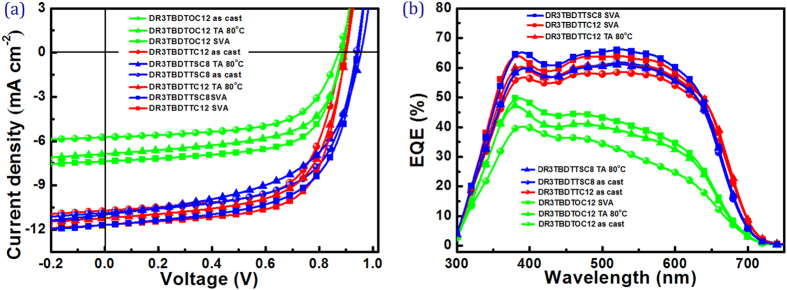
(**a**) *J–V* characteristics and (**b**) EQE curves of the devices based on SM:PC_71_BM (w:w, 1:1) as cast, 80 °C TA for 10 min and SVA with CF for 1 min.

BHJ solar cells were fabricated by spin-coating the chloroform solution of SMs and PC_71_BM onto PEDOT:PSS coated ITO glass and successive deposition of PFN and Al to make the device into structure of ITO/PEDOT:PSS/SM:PC_71_BM/PFN/Al. Characteristics of devices were investigated by measuring the corresponding current density-voltage (*J–V*) curves under the irradiation of AM 1.5 G (100 mW cm^−2^) solar simulator. Typical results are summarized in Table 2, while representative *J–V* curves are plotted in Fig. 5a. The weight ratio of SM and PC_71_BM (SM:PC_71_BM) in the active layer was firstly investigated to achieve good device performance. By increasing PC_71_BM content to adjust the weight ratio of SM:PC_71_BM from 1:0.5, 1:0.75, 1:1, 0.75:1 and 0.5:1, all three SM-OSCs present the maximal PCE values at SM:PC_71_BM weight ratio of 1:1. This weight ratio was thus adopted in the following device optimization. A close look at the device performance of three SMs under same conditions, one can find that 2-D BDT based SMs delivered much higher photovoltaic performance than DR3TBDTOC12 ones, which was consistent with their optical and electrochemical performance. The SM:PC_71_BM (1:1, w-w) devices contributed the maximal PCE of 3.11%, 6.10%, and 6.32% for **DR3TBDTOC12**, **DR3TBDTTC12** and **DR3TBDTTSC8**, respectively.

Compared with **DR3TBDTOC12** devices, **DR3TBDTTC12** and **DR3TBDTTSC8** based devices showed higher *V*_*oc*_ values, especially **DR3TBDTTSC8** exhibited the highest *V*_*oc*_ (~0.93 V). The rather high *V*_*oc*_ values of 2-D BDT SMs are attributed to their lower-lying HOMO level (Table 1). More importantly, the 2-D BDT based SM-OSCs exhibited much improved *J*_*sc*_ and FF values than **DR3TBDTOC12** devices, indicating the beneficial of conjugated side-chains in promoting anisotropic charge transportation in active layer^24^,^39^.

The SM:PC_71_BM devices were further optimized using thermal annealing (TA)^18^ and solvent vapor annealing (SVA)^10^ treatment. The photovoltaic parameters are summarized in Table 3. By annealing the active layer at 80 °C for 10 min, **DR3TBDTOC12** and **DR3TBDTTC12** based OSCs showed greatly improved PCEs, where *J*_*sc*_ exhibited dramatic improvement and *V*_*oc*_ presented slight enhancement. The FF values of all devices, however, decreased after TA treatment. **DR3TBDTTC12**:PC_71_BM devices after TA treatment showed a highest PCE of 6.37%, with a *J*_*sc*_ of 11.23 mA cm^−2^, a *V*_*oc*_ of 0.90 V, and a FF of 62.98%. **DR3TBDTTSC8** based devices, however, exhibited slightly decreased PCE after annealing, mainly attributed to the great decrease in FF value.

SVA with CF vapor was also explored for the optimization of SM-OSCs. All devices after SVA showed higher PCEs than devices as cast and annealed, mainly due to the improved *J*_*sc*_ values. **DR3TBDTTC12**:PC_71_BM treated by CF gave a PCE of 6.99%, while a PCE of 6.78% was obtained for **DR3TBDTTSC8** device. The **DR3TBDTOC12** devices treated by CF also gave a greatly increased PCE of 4.25%. Although **DR3TBDTTSC8** based OSC based displayed the highest *J*_*sc*_ of 11.70 mA cm^−2^ and *V*_*oc*_ of 0.94 V, the relatively low FF (61.61%) restricted the device performance. It should be noted that 2-D BDT cored SMs have better photovoltaic performance than 1-D BDT cored SM. The introduction of sulfur atom in side chain may afford devices with higher *V*_*oc*_ and *J*_*sc*_, providing great potential to achieve high performance OSCs.

To investigated the difference of *J*_*sc*_ values in the three BHJ SM-OSCs, the hole mobility of the molecules was measured by the method of organic field effect transistor (OFET). P **DR3TBDTOC12**, **DR3TBDTTC12** and **DR3TBDTTSC8** showed hole mobilities of 3.73 × 10^−5^ cm^2^ V^−1^ s^−1^, 1.02 × 10^−3^ cm^2^ V^−1^ s^−1^ and 7.25 × 10^−4^ cm^2^ V^−1^ s^−1^ and, respectively (Figure S1 in the Supporting Information). The one-magnitude lower hole mobility of **DR3TBDTOC12** could be attributed to its lower *J*_*sc*_ in BHJ cells in comparison to **DR3TBDTTC12** and **DR3TBDTTSC8**. The extended conjugation along side-chains of 2-BDT core is thus beneficial for higher hole transfer.

Figure 5b shows the external quantum efficiency (EQE) of the best SM-OSC devices under different treatments as a function of wavelength, which is consistent with the UV-vis absorption spectra of SM:PC_71_BM. Three SMs exhibited EQE spectra covering a broad absorption from 300 nm to 700 nm. The EQE spectrum of **DR3TBDTTSC8** and **DR3TBDTTC12** extends further to the red than **DR3TBDTOC12**, consistent with the insertion of thienyl and thiothienyl side chain leading to extended red absorption in these SMs as shown in Fig. 2c. Convolution of the EQE spectrum with AM1.5 solar spectrum^18^,^19^ afforded the calculated *J*_*sc*_ in good good agreement (±0.5 mA cm^−2^) with those measured (7.38, 11.69, and 11.70 mA cm^−2^) under AM1.5 simulated sunlight (Table 3).

In comparison, **DR3TBDTTSC8** and **DR3TBDTTC12** based devices show 10~20% higher EQE values than **DR3TBDTOC12** in a wide wavelength range of 350~700 nm, which result in higher *J*_sc_ values in 2-D BDT based SMs devices. Especially, **DR3TBDTTSC8** based devices exhibit EQE over 60% in wavelength band ranging from 380 to 650 nm with SVA treatment. In comparison, **DR3TBDTOC12** based devices is below 50% and it shows much lower intensity in the range of 450~700 nm, which is related to the intermolecular charge transfer (ICT) between D and A units of SMs. These results are echoing the results of UV-vis absorption and prove that the incorporation of conjugated side chains onto BDT core could effectively intensify the EQE response and thus significantly improve the photoconversion efficiency for the devices.

**Figure 6:**
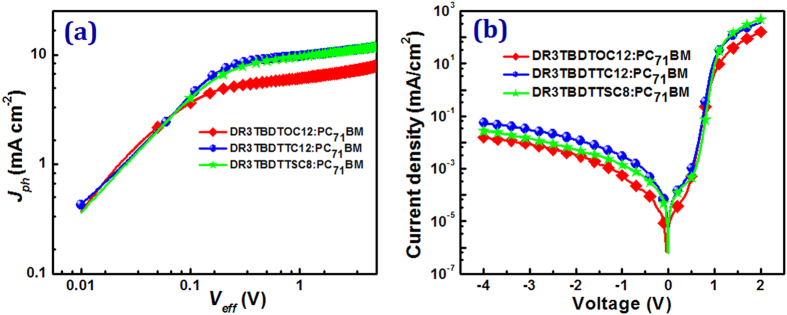
(**a**) The *J*_*ph*_ − *V*_*eff*_ curves and (**b**) dark *J–V* characteristic curves of SMs:PC_71_BM(1:1, w:w) OSCs treated with CF vapor annealing.

To gain the insight into the mechanism responsible for the improved performance for the SM-OSCs with SVA treatment, the photocurrent versus effective voltage (*J*_*ph*_ − *V*_*eff*_) curves and the dark *J*–*V* characteristic curves has been investigated and shown in Fig. 6. All devices showed low dark current density under the reverse bias, indicating an effectively restrain of leakage current, which may provide effective charge carriers transport in blend layers^40^,^41^. As we know, the *J*_*ph*_ is defined as the difference between the current density under illumination (*J*_*L*_) and the current density in the dark (*J*_*D*_), thus *J*_*ph*_ = *J*_*L*_ − *J*_*D*_. *V*_*eff*_ is defined as *V*_*eff*_ = *V*_*o*_ − *V*_*a*_. *V*_*o*_ is the voltage at which *J*_*ph*_ = 0 and *V*_*a*_ is the applied bias voltage^18^,^42^. Compared to devices based on **DR3TBDTOC12** and **DR3TBDTTC12**, it was apparent that *J*_*ph*_ value of **DR3TBDTTSC8** based device showed a stronger field dependence. SM-OSCs based on **DR3TBDTOC12** and **DR3TBDTTC12** showed higher *J*_*ph*_ and rapidly reached a saturation state (*J*_*sat*_) at relatively low *V*_*eff*_ (ca. 0.23 V), while 0.38 V for **DR3TBDTTSC8** based device. These results suggest a more severely geminate and/or bimolecular recombination and/or less efficient interfacial contact occurring at **DR3TBDTTSC8** devices, thus leading to a lower FF^18^,^42^,^43^.

### Morphology

**Figure 7:**
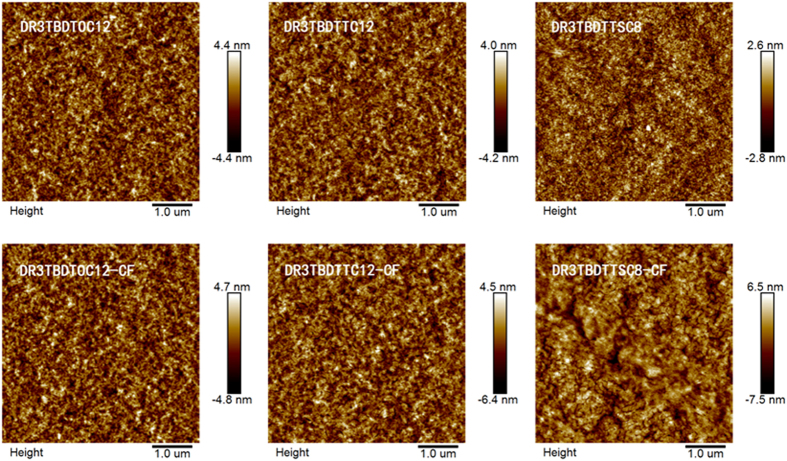
AFM morphology of SM:PC_71_BM blend films as cast (top panel) and after CF vapor annealing (bottom panel).

To further understand the enhanced photovoltaic performance of SM-OSCs with CF vapor annealing, the surface topography images of SM:PC_71_BM(1:1, w/w) blend films have been observed using tapping-mode AFM (Fig. 7). Networks of fibrils with diameters of ~10 nm (commensurating with the exciton diffusion length^13^) are observed for all SM:PC_71_BM blend films, which favour high exciton diffusion/dissociation efficiency in the active layers of OSCs^44^. Upon SVA, more pronounced difference is observed for **DR3TBDTTSC8**:PC_71_BM films, though fibrillar network morphology was retained and even strengthened for the rest two blend films. The phase-separated morphology of **DR3TBDTTSC8**:PC_71_BM blend significantly coarsened, with larger domain size (40~80 nm) observed. Thus, **DR3TBDTTSC8**:PC_71_BM blend may have higher probability of exciton recombination before reaching the donor–acceptor interface, resulting in low FF for the device.

The root-mean-square (RMS) roughness of the surface of blend films as cast is 1.23, 1.17 and 0.762 nm for **DR3TBDTOC12**:PC_71_BM**, DR3TBDTTC12**:PC_71_BM, and **DR3TBDTTSC8**:PC_71_BM, respectively. After SVA with CF, the RMS roughness of surface for the corresponding blend films increased to 1.33, 1.53, and 1.93 nm. Although the RMS roughness slightly increased after CF vapor annealing, the performance of devices still improved, that may be ascribed to the improvement of the miscibility in films, a more continuous and homogeneous network could be seen in the photographs^45^,^46^.

Further device optimization by screening appropriate solvents for SVA is under way. For polymer OSCs, it is reported that SVA can fine-tuning the morphology in the active layer due to the penetration of solvent vapor into polymer/fullerene blends to lower the glass transition temperature of polymer to facilitate the morphology evolution. Hole mobility was improved for the polymer/fullerene blends^44^. When applying SVA for molecule OSCs, the morphology evolution will be mainly determined by phase separation and molecule crystallization and growth. The systematic study of morphology change with SVA treatment revealed that the presence of solvent molecules inside the BHJ thin film promotes the mobility of both donor and acceptor molecules, leading to crystallization and aggregation, which are important in modulating the thin film morphology^10^.

## Conclusion

Three novel SMs based on alkoxy, alkylthienyl and alkylthiothienyl substituted BDT core unit were designed for photovoltaic application. This side-chain engineering exerts subtle but measurable measurable effect on the UV absorption, bandgaps and energy levels of the SMs. The XRD analysis and molecular simulation revealed that the conjugated side chains facilitated densely packing along interchains for 2-D BDT cored SMs. The pristine SM-OSCs with a structure of ITO/PEDOT:PSS/SM:PC_71_BM/PFN/Al obtained a PCE of 3.11%, 6.10% and 6.32%, respectively, without any treatment. Thermal annealing and chloroform vapor annealing were effective approaches device optimization. The device PCEs were improved to 4.25%, 6.99% and 6.78% for three SM-OSCs after solvent vapor annealing. The introduction of sulfur atom in side chain of BDT afforded the OSCs with higher *V*_*oc*_ and *J*_*sc*_ values. 2-D BDT based SMs with sulfur atom in side chain were demonstrated as good candidate for high performance OSCs.

## Additional Information

**How to cite this article**: Yin, X. *et al.* Side-chain Engineering of Benzo[1,2-b:4,5-b’]dithiophene Core-structured Small Molecules for High-Performance Organic Solar Cells. *Sci. Rep.* **6**, 25355; doi: 10.1038/srep25355 (2016).

## Supplementary Material

Supplementary Information

## Figures and Tables

**Table 1:** Optical and electrochemical data of SMs.

Molecule	*λ*_*max*_(nm)^a^	*λ*_*max*_(nm)^b^	*λ*_*onset*_(nm)^b^	*E*_*g*_^*opt*^ (eV)^c^	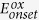 (eV)	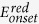 (eV)	HOMO (eV)^*d*^	LUMO (eV)^*d*^	*E*_*g*_^*cv*^ (eV)^*d*^
**DR3TBDTOC12**	502	571,612	703	1.76	1.01	−0.72	−5.32	−3.59	1.73
**DR3TBDTTC12**	505	582,627	707	1.75	1.03	−0.68	−5.34	−3.63	1.71
**DR3TBDTTSC8**	504	583,628	716	1.73	1.07	−0.66	−5.38	−3.65	1.73

^a^Solution;

^b^Film;

^c^*E*_*g*_^*opt*^ = 1240/*λ*_*onset*_ (eV); HOMO = 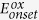 + 4.31 (eV), LUMO = 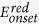 + 4.31 (eV), *E*_*g*_^*cv*^ = 
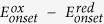
.

**Table 2:** Photovoltaic performance of SM:PC_71_BM devices at different weight ratios.

Material	SM:PC_71_BM	*V*_oc_ [V]	*J*_sc_ [mA cm^−2^]	FF [%]	PCE (ave.) [%]^a^
**DR3TBDTOC12**	1:0.5	0.87	3.95	52.90	1.82 [1.75]
	1:0.75	0.87	5.47	60.19	2.86 [2.74]
	**1:1**	**0.87**	**5.74**	**62.18**	**3.11 [3.07]**
	0.75:1	0.83	4.51	48.02	1.80 [1.74]
	0.5:1	0.81	4.19	43.83	1.48 [1.43]
**DR3TBDTTC12**	1:0.5	0.89	10.33	58.07	5.34 [5.25]
	1:0.75	0.89	10.47	60.83	5.67 [5.59]
	**1:1**	**0.89**	**10.73**	**63.92**	**6.10 [5.48]**
	0.75:1	0.89	10.09	53.05	4.76 [4.68]
	0.5:1	0.85	6.77	39.47	2.27 [2.20]
**DR3TBDTTSC8**	1:0.5	0.93	7.82	50.48	3.67 [3.59]
	1:0.75	0.94	11.01	55.45	5.74 [5.68]
	**1:1**	**0.94**	**10.88**	**61.77**	**6.32 [6.24]**
	0.75:1	0.93	10.01	48.06	4.47 [4.39]
	0.5:1	0.92	7.81	40.24	2.89 [2.81]

^a^Average values of ten devices.

**Table 3:** Photovoltaic performance of SM:PC_71_BM (1:1, w/w) OSCs with different treatment.

Material	Treatment	*V*_oc_ [V]	*J*_sc_ [mA cm^−2^]	FF [%]	PCE [%]^a^
**DR3TBDTOC12**	As cast	0.87	**5.74**	**62.18**	**3.11 [3.07]**
	80 °C annealing	0.89	6.89	60.82	3.73 [3.68]
	**CF vapor annealing**	**0.88**	**7.38**	**65.39**	**4.25 [4.18]**
**DR3TBDTTC12**	As cast	0.89	**10.73**	**63.92**	**6.10 [5.48]**
	80 °C annealing	0.90	11.23	62.98	6.37 [6.32]
	**CF vapor annealing**	**0.90**	**11.69**	**66.48**	**6.99 [6.92]**
**DR3TBDTTSC8**	As cast	0.94	**10.88**	**61.77**	**6.32 [6.24]**
	80 °C annealing	0.96	11.01	54.09	5.72 [5.65]
	**CF vapor annealing**	**0.94**	**11.70**	**61.61**	**6.78 [6.69]**

^a^Average values of ten devices.
