# Supplementary material for: Side-chain Engineering of Benzo[1,2-b:4,5-b’]dithiophene Core-structured Small Molecules for High-Performance Organic Solar Cells
Source: Sci Rep. 2016 May 3;6:25355. doi: 10.1038/srep25355 (PMC4853745; doi:10.1038/srep25355)
Supplement: Supplementary Information [file srep25355-s1.doc]

Supporting Information

Side-chain Engineering of Benzo[1,2-b:4,5-b’]dithiophene Core-structured Small Molecules for High-Performance Organic Solar Cells

Xinxing Yin^1,4^, Qiaoshi An^2,4^, Jiangsheng Yu^1^, Fengning Guo^3^, Linyi Bian^3^, Yongliang Geng^1^, Zhongsheng Xu^1^, Baojing Zhou^1^, Linghai Xie^3^, Fujun Zhang^2^ and Weihua Tang^1^

^a^ Key Laboratory of Soft Chemistry and Functional Materials (Ministry of Education of China), Nanjing University of Science and Technology, Nanjing, 210094, China

^b^ Key Laboratory of Luminescence and Optical Information (Ministry of Education of China), Beijing Jiaotong University, Beijing, 100044, China

^c^ Institute of Advanced Materials, Nanjing University of Posts and Telecommunications, Nanjing, 210023, China

**1. Material synthesis**

All chemicals (reagent grade) were purchased from Sigma-Aldrich Co. and used without further purification. Toluene, tetrahydrofuran (THF), 1,2-dichloroethane and chloroform were freshly distilled before use. Other solvents were used directly.

**3-Octylthiophene (1).** A Grignard reagent was freshly prepared with the following steps. To a mixture of magnesium ribbon (3.46 g, 142.40 mmol) and 3 grains of I_2_ in freshly dried THF (5 mL), a solution of 1-bromooctane (25g, 129.45 mmol) in THF (15 mL) was added dropwise. After stirring at room temperature for 1 h, the mixture was heated to reflux for another 1 h. The Grignard reagent was then added slowly into the mixture solution of 3-bromothiophene (17 g, 104.27 mmol) and Ni(dppp)_2_Cl_2_ in THF (150 mL) under N_2_ protection. The reaction mixture was heated to reflux overnight. After cooling to room temperature, the solution was quenched with aq. HCl (100 mL, 1 M) solution. Extracted with ethyl acetate (EA) and washed with water and brine, the combined organic phase was dried over by MgSO_4_ and concentrated *in vacuo*. The residue was purified by flash column chromatography with hexane to afford the title compound as a colorless oil (16 g, 78%). ^1^H NMR (500 MHz, CDCl_3_, δ/ppm): 7.24 (dd, *J* = 4.9, 2.9 Hz, 1H), 6.94 (d, *J* = 4.9 Hz, 1H), 6.92 (dd, *J* = 2.8, 0.8 Hz, 1H), 2.78 – 2.40 (m, 2H), 1.62 (dd, *J* = 14.7, 7.1 Hz, 2H), 1.40 – 1.18 (m, 10H), 0.89 (dd, *J* = 7.0, 6.5 Hz, 3H).

**2-Bromo-3-octylthiophene (2).** Towards the solution of **1** (10 g, 50.93 mmol) in THF (100 mL) in ice bath was added N-bromosuccinimide (NBS, 9.06 g, 50.93 mmol) in portions. The mixture solution was then stirred overnight. After extracted with EA, washed with water and brine, the organic layer was dried with MgSO_4_. After removal of solvent, the crude compound was purified by column chromatography on silica gel with hexane to afford compound **2** (13 g, 93%) as a colorless oil. ^1^H NMR (500 MHz, CDCl_3_, δ/ppm): 7.18 (d, *J* = 5.6 Hz, 1H), 6.79 (d, *J* = 5.6 Hz, 1H), 2.61–2.53 (m, 2H), 1.58 (m, 2H),1.38–1.12 (m, 10H), 0.88 (t, *J* = 6.9 Hz, 3H).

**2,5-Bis(trimethylstannyl)thiophene (3).** To a mixture solution of thiophene (1.50 g, 17.83 mmol) and TMEDA (5.38 mL, 35.66 mmol) in dry THF (50 mL) at 0°C was added with *n*-BuLi (16.3 mL, 39.22 mmol, 2.40 M in *n-*hexane). The reaction mixture was heated to reflux for 1 h and cooled to 0°C again before the addition of a solution of trimethyltin chloride (8.53 g, 42.79 mmol) in THF (20 mL). After removal of the cooling bath, the mixture was allowed to stir at room temperature overnight. Quenched with saturated ammonium chloride solution (50 mL), the reaction mixture was extracted with diethyl ether and washed with brine twice. Dried over MgSO_4_, the organic layer was concentrated *in vacuo* to afford compound **3** (4.4 g, 60%) as a white crystal. ^1^H NMR (500 MHz, CDCl_3_, δ/ppm): 7.37 (s, 2H), 0.36 (s, 18H).

**3,3''-Dioctyl-2,2':5',2''-terthiophene (4).** To the mixture solution of **2** (5.91 g, 21.48 mmol) and **3** (4 g, 9.76 mmol) in dry toluene (100 mL) was added with the catalyst, Pd(PPh_3_)_4_ (562.75 mg, 0.49 mmol). The solution was refluxed for 16 h under N_2_ protection. After cooled to room temperature, the reaction mixture was concentrated under reduced pressure. The crude mixture was purified by column chromatography on silica gel using hexane as eluent. The title compound **4** (3.2 g, 69%) was obtained as a bright yellow liquid. ^1^H NMR (500 MHz, CDCl_3_, δ/ppm): 7.17 (d, *J* = 5.2 Hz, 2H), 7.05 (s, 2H), 6.94 (d, *J* = 5.2 Hz, 2H), 2.80–2.75 (m, 4H), 1.70–1.60 (m, 4H), 1.36 – 1.28 (m, 20H), 0.87 (t, *J* = 6.9 Hz, 6H).

**3,3''-Dioctyl-[2,2':5',2''-terthiophene]-5-carbaldehyde (5).** To a solution of **4** (3 g, 6.35 mmol) and DMF (463.78 mg, 6.35 mmol) in 1,2-dichloroethane, POCl_3_ was slowly added at 0°C under N_2_. After stirring for 1 h, the mixture was heated at 60 °C overnight. The reaction mixture was cooled to 0°C and then neutralized with saturated NaHCO_3_ solution. The organic phase was extracted with CH_2_Cl_2_, washed with water and brine. Dried over MgSO_4_, the organic layer was concentrated *in vacuo*. The residue was purified by column chromatography (hexane/CH_2_Cl_2_, v/v, 5/1) to give compound **5** (3 g, 94%) as a yellow oil. ^1^H NMR (500 MHz, CDCl_3_, δ/ppm): 9.83 (s, 1H), 7.60 (s, 1H), 7.24 (d, *J* = 3.8 Hz, 1H), 7.22 (d, *J* = 5.2 Hz, 1H), 7.10 (d, *J* = 3.8 Hz, 1H), 6.96 (d, *J* = 5.2 Hz, 1H), 2.85–2.76 (m, 4H), 1.78–1.60 (m, 4H), 1.48–1.17 (m, 20H), 0.87 (t, *J* = 6.9, 3.3 Hz, 6H).

**5''-Bromo-3,3''-dioctyl-[2,2':5',2''-terthiophene]-5-carbaldehyde (6).** To a vigorously stirred solution of **5** (3 g, 5.99 mmol) in THF (50 mL) in ice-water bath was added with NBS (1.17 g, 6.59 mmol). The reaction solution was stirred at room temperature overnight. Extracted with CH_2_Cl_2_, the organic layer was further washed with water and brine before dried over with MgSO_4_. After removal of solvent, the residue was purified by column chromatography on silica gel (hexane/CH_2_Cl_2_, v/v, 3/1) to afford *the title* compound (2.8g, 81%) as a brown oil. ^1^H NMR (500 MHz, CDCl_3_, δ/ppm): 9.83 (s, 1H), 7.60 (s, 1H), 7.22 (d, *J* = 3.8 Hz, 1H), 7.04 (d, *J* = 3.8 Hz, 1H), 6.92 (s, 1H), 2.83–2.79 (m, 2H), 2.75–2.67 (m, 2H), 1.68 (d, *J* = 7.6 Hz, 2H), 1.61 (d, *J* = 7.6 Hz, 2H), 1.46–1.16 (m, 20H), 0.87 (t, *J* = 6.8, 4.0 Hz, 6H).

**4,8-Dihydroxybenzo[1,2-b:4,5-b']dithiophene (I).** To a stirred suspension of 4,8-dihydrobenzo[1,2-b:4,5-b']dithiophen-4,8-dione (15 g, 68.10 mmol) in ethanol (250 mL) at 0^o^C was added with NaBH_4_ (5.67 g, 149.82 mmol) in portions. The reaction mixture was stirred at 85°C for 12 h. The reaction was quenched by pouring the reaction mixture into aq. HCl (100 mL, 1 M) solution. The crude product was filtered and washed with water. After dried at 70°C *in vacuo*, the title compound **I** was obtained as a green solid (14.90 g, 99%) without further purification. ^1^H NMR (500 MHz, DMSO-_6_, δ/ppm): 9.80 (s, 2H), 7.60 (d, *J* = 5.6 Hz, 2H), 7.54 (d, *J* = 5.5 Hz, 2H).

**4,8-Bis(dodecyloxy)benzo[1,2-b:4,5-b']dithiophene (II).** To a suspension of **I** (1 g, 4.50 mmol) and K_2_CO_3_ (6.22 g, 45.00 mmol) in DMF (50 ml), 1-bromododecane (3.36 g, 13.50 mmol) was added and the reaction mixture was stirred at 80°C for 24h. The organic phase was extracted with CH_2_Cl_2_, washed with water and brine, dried over MgSO_4_. After removal of solvent, the residue was was recrystallized from ethanol to give the compound **II** (2.2 g, 85%) as a colorless solid. ^1^H NMR (500 Hz, CDCl_3_, δ/ppm): 7.48 (d, *J* = 5.5 Hz, 2H), 7.36 (d, *J* = 5.2 Hz, 2H), 4.27 (t, *J* = 6.6 Hz, 4H), 1.91**-**1.84 (m, 4H), 1.60**-**1.52 (m, 4H), 1.42**-**1.22 (m, 32H), 0.88 (t, *J* = 6.9 Hz, 6H).

**2,6-Bis(trimethylstannane)-4,8-bis(dodecyloxy)benzo[1,2-b:4,5-b']dithiophene (7).** *n*-BuLi (1.64 mL, 3.94 mmol, 2.40 M in hexane) was added dropwise to the solution of **II** (1 g, 1.79 mmol) in dry THF (50 mL) at **-**78 °C. After stirred at **-**78 °C for 2h, a solution of trimethyltin chloride (0.89 g, 4.47 mmol) in THF (5 mL) was added. The reaction mixture was gradually warmed to room temperature and stirred overnight. The reaction was quenched with water (50 mL). Extracted with diethyl ether, the organic layer was washed with brine twice. After dried over MgSO_4_, the organic layer was concentrated *in vacuo*. The residue was recrystallized from isopropanol to yield the compound **7** as colorless needles (1.22 g, 77%). ^1^H NMR (500 MHz, CDCl_3_, δ/ppm): 7.51 (s, 2H), 4.29 (t, *J* = 6.5 Hz, 4H), 1.92–1.84 (m, 4H), 1.62–1.54 (m, 4H), 1.42–1.24 (m, 32H), 0.88 (t, *J* = 6.8 Hz, 6H), 0.45 (s, 18H).

**4,8-Bis(trifluoromethanesulfonyloxy)benzo[1,2-b:4,5-b']dithiophene(III).** To a suspension of **I** (10 g, 45 mmol) and dry pyridine (10.87 mL) in dichloromethane (200 mL) was slowly added trifluoromethanesulfonic anhydride (22.67 mL, 134.96 mmol) at 0°C. After stirring at 0°C for 12 h, water (100 mL) and hydrochloric acid (150 mL, 1 M) were added. The resulting mixture was extracted with dichloromethane. The combined organic layer was dried over MgSO_4_ and concentrated *in vacuo.* The residue was purified with column chromatography on silica gel with petroleum ether/ethyl acetate (19:1) as eluent to give compound **III** as a white solid (17.2 g, 78%). ^1^H NMR (500 MHz, DMSO-_6_, δ/ppm): 8.20 (d, *J* = 5.6 Hz, 2H), 7.55 (d, *J* = 5.5 Hz, 2H).

**4,8-Bis(5-dodecylthiophen-2-yl)benzo[1,2-*b*:4,5-*b’*]dithiophene (IV).** Compound **III** (1 g, 2.06 mmol), Pd(PPh_3_)_4_ (118.79 mg, 0.1 mmol), 2-(5-dodecylthiophen-2-yl)-4,4,5,5- tetramethyl-1,3,2-dioxaborolane (2.33 g, 6.17 mmol) were dissolved in THF (50 mL, degassed with nitrogen) and aqueous sodium carbonate (20 mL, 1.0 M, degassed with nitrogen). The mixture was heated at 85°C for 24 h and then poured onto aqueous HCl solution. The mixture was extracted with dichloromethane and washed with water. The organic phase was dried and concentrated. The residue was purified by column chromatography on silica gel using petroleum ether as eluent to afford the title product as a yellow solid (1.02 g, 72%). ^1^H NMR (500 MHz, CDCl_3_, δ/ppm): 7.65 (d, *J* = 5.7 Hz, 2H), 7.46 (d, *J* = 5.7 Hz, 2H), 7.30 (d, *J* = 3.5 Hz, 2H), 6.92 (d, *J* = 3.5 Hz, 2H), 2.93 (t, *J* = 7.7 Hz, 4H), 1.83**-**1.75 (m, 4H), 1.50**-**1.42 (m, 4H), 1.40**-**1.27 (m, 32H), 0.89 (t, *J* = 6.9 Hz, 6H).

**2,6-Bis(trimethyltin)-4,8-bis(5-dodecylthiophen-2-yl)benzo[1,2-*b*:4,5-*b’*]dithiophene (8).** Compound **IV** (1 g, 1.45 mmol) and anhydrous THF (50 mL) were added into a ﬂask under nitrogen atmosphere. The solution was cooled to 0°C and *n*-BuLi (1.5 mL, 3.62 mmol, 2.4 M in hexane) was added dropwisely. The reaction mixture was then stirred for 2 h at room temperature and cooled to 0 °C. Subsequently, 3.6 mL of trimethyltin chloride (3.62 mmol, 1.0 M in THF) was added in one portion and the mixture was stirred at room temperature overnight. The mixture was quenched by addition of 20 mL of water and extracted with diethyl ether three times. The combined organic phase was dried with MgSO_4_, filtered and concentrated via rotary evaporation. The residue was recrystallized in isopropanol to obtain compound **8** as a light yellow solid (1.40 g, 1.38 mmol, 95%). ^1^H NMR (500 MHz, CDCl_3_, δ/ppm): 7.68 (s, 2H), 7.31 (d, *J* = 3.5 Hz, 2H), 6.92 (d, *J* = 3.5 Hz, 2H), 2.93 (t, *J* = 7.7 Hz, 4H), 1.83**-**1.74 (m, 4H), 1.50**-**1.41 (m, 4H), 1.39**-**1.26 (m, 32H), 0.88 (t, *J* = 6.9 Hz, 6H), 0.39 (s, 18H).

**4,4,5,5-Tetramethyl-2-(5-(octylthio)thiophen-2-yl)-1,3,2-dioxaborolane (V).** Towards a solution of 2-(octylthio)thiophene (3 g, 13.13 mmol) in THF (100 mL) at -78°C were added *n*-BuLi (6.6 mL, 15.76 mmol, 2.4 M in hexane) under nitrogen atmosphere. The reaction mixture was then stirred for 2 h at room temperature and cooled to -78 °C. Subsequently, 2-isopropoxy-4,4,5,5-tetramethyl-1,3,2-dioxaborolane (5.38 g, 28.89 mmol) was added in one portion. The mixture was stirred at room temperature overnight. Quenched with water (50 mL), the mixture was extracted with diethyl ether three times. The combined organic phase was dried with Na_2_SO_4_ and concentrated. The residue was purified with column chromatography on silica gel with petroleum ether/ethyl acetate (20:1) as eluent to afford compound **V** (3.3 g, 71%) as a purple oil. ^1^H NMR (500 MHz, DMSO-_6_, δ/ppm): 7.43 (dd, *J* = 3.6, 1.4 Hz, 1H), 7.17 (dd, *J* = 3.7, 1.4 Hz, 1H), 2.90 (t, *J* = 7.3 Hz, 2H), 1.63–1.47 (m, 2H), 1.43–1.07 (m, 22H), 0.95–0.74 (m, 3H). ^13^C NMR (126 MHz, DMSO-_6_, δ/ppm): 143.26, 138.36, 132.46, 84.53, 37.48, 31.61, 29.17, 28.97, 28.84, 28.15, 24.98, 22.51, 14.39.

**4,8-Bis(5-(octylthio)thiophen-2-yl)benzo[1,2-b:4,5-b']dithiophene (VI).** Compound **III** (1.5 g, 3.08 mmol), **V** (3.28 g, 9.25 mmol), and Pd(PPh_3_)_4_ (178.18mg, 0.15 mmol) were dissolved in THF (50 mL, degassed with nitrogen) and aqueous sodium carbonate (20 mL, 1.0 M, degassed with nitrogen). The mixture was heated at 85°C for 24 h before poured into aqueous HCl solution. Extracted with dichloromethane, the organic phase was washed with water, dried over Na_2_SO_4_ and concentrated. The yellow product **IV** (1.2 g, 61%) was obtained by column chromatography on silica gel using petroleum ether. ^1^H NMR (500 MHz, CDCl_3_, δ/ppm): 7.62 (d, *J* = 5.7 Hz, 2H), 7.48 (d, *J* = 5.7 Hz, 2H), 7.34 (d, *J* = 3.6 Hz, 2H), 7.22 (d, *J* = 3.6 Hz, 2H), 2.96–2.88 (m, 4H), 1.72 (tt, *J* = 7.5, 6.4 Hz, 4H), 1.50–1.40 (m, 4H), 1.37–1.21 (m, 14H), 0.94–0.83 (m, 6H).

**2,6-Bis(trimethyltin)-4,8-bis(5-dodecylthiophen-2-yl)benzo[1,2-*b*:4,5-*b’*]dithiophene (9).** Towards a solution of compound **VI** (1 g, 1.55 mmol) in THF (50 mL) at -78 °C was added with *n*-BuLi (1.43 mL, 3.42 mmol, 2.4 M in hexane). The reaction mixture was then stirred for 2 h at room temperature and cooled to -78°C. Subsequently, trimethyltin chloride (3.89 mL, 3.89 mmol, 1.0 M in THF) was added in one portion. The mixture was stirred at room temperature overnight. The mixture was quenched by the addition of 50 mL water. Extracted with diethyl ether, the combined organic phase was dried with MgSO_4_ and concentrated via rotary evaporation. The residue was recrystallized with isopropanol to afford *the title* compound as a yellow solid (1.48 g, 98%). ^1^H NMR (500 MHz, CDCl_3_, δ/ppm): 7.66 (s, 2H), 7.37 (d, *J* = 3.6 Hz, 2H), 7.24 (d, *J* = 3.6 Hz, 2H), 2.98–2.89 (m, 4H), 1.79–1.68 (m, 4H), 1.46 (td, *J* = 8.7, 4.5 Hz, 4H), 1.40–1.16 (m, 14H), 0.97–0.81 (m, 6H), 0.41 (s, 18H).

**5'',5'''''-(4,8-Bis(dodecyloxy)benzo[1,2-b:4,5-b']dithiophene-2,6-diyl)bis(3,3''-d-ioctyl-[2,2':5',2''-Terthiophene]-5-carbaldehyde) (10).** A solution of compound **6** (300 mg, 0.52 mmol) and **7** (217.97 mg, 0.25 mmol) in dry toluene (20 mL) was degassed twice with N_2_, then Pd(PPh_3_)_4_ (28.48 mg, 0.025 mmol) was added. After stirring at 110°C for 24 h under N_2_, the mixture was cooled to room temperature. After removal of toluene, the crude product was purified by column chromatography on silica gel (hexane/CH_2_Cl_2_, 3/1, v/v,) to afford compound **10** (200 mg, 52%) as a red solid. ^1^H NMR (500 MHz, CDCl_3_, δ/ppm): 9.83 (s, 2H), 7.60 (s, 2H), 7.47 (s, 2H), 7.25 (s, 2H), 7.15 (d, *J* = 3.2 Hz, 4H), 4.29 (t, *J* = 6.5 Hz, 4H), 3.00–2.64 (m, 8H), 2.00–1.85 (m, 4H), 1.77–1.66 (m, 8H), 1.59 (dd, *J* = 15.0, 7.4 Hz, 4H), 1.50–1.40 (m, 12H), 1.40–1.17 (m, 60H), 0.94–0.78(m, 18H). ^13^C NMR (126 MHz, CDCl_3_, δ/ppm): 182.51, 143.96, 141.19, 140.93, 140.41, 140.32, 139.03, 137.86, 136.20, 135.66, 134.81, 132.59, 130.25, 129.40, 128.38, 127.85, 126.34, 116.23, 73.96, 31.92, 30.55, 30.32, 29.70, 29.51, 29.32, 26.08, 22.70, 14.12. MALDI-TOF MS: calcd. for C_84_H_114_O_4_S_8_ *m/z* = 1442.65; found 1442.77.

**5'',5'''''-(4,8-Bis(5-dodecylthiophen-2-yl)benzo[1,2-b:4,5-b']dithiophene-2,6-di-yl)bis(3,3''-dioctyl-[2,2':5',2''-terthiophene]-5-carbaldehyde) (11).** By adopting similar synthetic approach as for **10**, compound **11** was prepared by the Pd-catalyzed Suzuki coupling between compound **6** (300 mg, 0.52 mmol) and **8** (250.56 mg, 0.25mmol) as a red solid with 60% yield. ^1^H NMR (500 MHz, CDCl_3_, δ/ppm): 9.83 (s, 2H), 7.63 (s, 2H), 7.60 (s, 2H), 7.31 (d, *J* = 3.4 Hz, 2H), 7.25 (d, *J* = 3.9 Hz, 2H), 7.13 (d, *J* = 5.8 Hz, 4H), 6.96 (d, *J* = 3.2 Hz, 2H), 2.96 (t, *J* = 7.6 Hz, 4H), 2.85–2.81 (m, 4H), 2.79 – 2.74 (m, 4H), 1.86–1.76 (m, 4H), 1.69 (dd, *J* = 15.1, 7.4 Hz, 8H), 1.52–1.45 (m, 4H), 1.30 (dd, *J* = 42.9, 28.7 Hz, 72H), 0.86 (dd, *J* = 7.0, 3.2 Hz, 18H). ^13^C NMR (126 MHz, CDCl_3_, δ/ppm): 182.43, 147.41, 141.00, 140.26, 140.17, 139.02, 138.66, 137.92, 137.33, 136.57, 135.62, 134.64, 130.26, 128.33, 127.94, 127.75, 126.13, 124.37, 123.31, 119.12, 31.92, 31.61, 30.45, 30.35, 30.27, 29.71, 29.53, 29.45, 29.31, 22.70, 14.13. MALDI-TOF MS: calcd. for C_92_H_118_O_2_S_10_ *m/z* = 1575.64; found 1575.81.

**5'',5'''''-(4,8-Bis(5-(octylthio)thiophen-2-yl)benzo[1,2-b:4,5-b']dithiophene-2,6-diyl)bis(3,3''-dioctyl-[2,2':5',2''-terthiophene]-5-carbaldehyde) (12).** By adopting similar synthetic approach as for compound **10**, the Pd-catalyzed Suzuki coupling between compound **6** (300 mg, 0.52 mmol) and **9** (238.71 mg, 0.25 mmol) afforded *the title* compound **12** (200 mg, 49%) as a red solid. ^1^H NMR (500 MHz, CDCl_3_, δ/ppm): 9.80 (s, 2H), 7.57 (s, 2H), 7.53 (s, 2H), 7.34 (d, *J* = 3.6 Hz, 2H), 7.27 (s, 2H), 7.21 (d, *J* = 3.7 Hz, 2H), 7.11 – 7.03 (m, 4H), 2.99 (dd, *J* = 9.8, 4.9 Hz, 4H), 2.87–2.77 (m, 4H), 2.73 (dd, *J* = 13.9, 6.5 Hz, 4H), 1.83–1.73 (m, 4H), 1.73–1.58 (m, 8H), 1.56–1.44 (m, 4H), 1.45–1.14 (m, 56H), 0.96–0.77 (m, 18H). ^13^C NMR (126 MHz, CDCl_3_, δ/ppm): 182.42, 141.82, 141.05, 140.85, 140.30, 138.99, 138.56, 137.76, 137.22, 137.11, 135.23, 134.77, 132.79, 130.57, 130.34, 128.50, 127.74, 126.21, 122.74, 118.62, 77.27, 77.02, 76.77, 58.25, 38.87, 31.88, 31.80, 30.47, 30.26, 29.71, 29.53, 29.44, 29.30, 29.22, 28.69, 28.57, 23.04, 22.68, 14.11. MALDI-TOF MS: calcd. for C_84_H_102_O_2_S_12_ *m/z* = 1526.45; found 1526.59.

**2. Hole mobility measurement from organic field effect transistor (OFET)**

For the carrier mobility measurement, OFET devices were made by a bottom-contact geometry. The source and drain contacts consisted of Cu (50 nm), and the dielectric was silicon oxide (SiO_2_) with a thickness of 300 nm. The SiO_2_ surface was ultrasonic cleaned sequentially with acetone, ethanol, and deionized water, and then treated in an ultraviolet-ozone chamber after drying in an oven. Then, polystyrene (PS) solution (3 mg ml^-1^) in toluene was spin-coated on the cleaned Si substrate at 3000 rpm for 30 seconds to a thickness of 12 nm, then the substrates were transferred in the oven to bake for 30 min at 80^o^C to produce smooth surfaces onto which the molecules could be spin-coated. The small molecules were dissolved in toluene with a concentration of 3mg/ml prior to use. Films of the organic semiconductors were spin-coated at 3000 rpm for 30 s to a thickness of about 50 nm, followed by an annealing process. Finally, Cu was thermally evaporated through a shadow mask to form source and drain electrodes with the channel width (W= 2000 μm) and the channel length (L= 100 μm). The measurement was carried out in ambient conditions using Keithley 4200 semiconducting parameter analyzer. The mobility of the devices was calculated on the saturated region according to the expression I_DS_ =(W/2L)μC_i_(V_G_-V_Th_)^2^, where I_DS_ is the drain-source current, μ is the field-effect mobility, and C_i_ is the capacitance (with a value of 10.6 in the experiment). V_G_ and V_th_ are the gate voltage and threshold voltage, respectively.

**
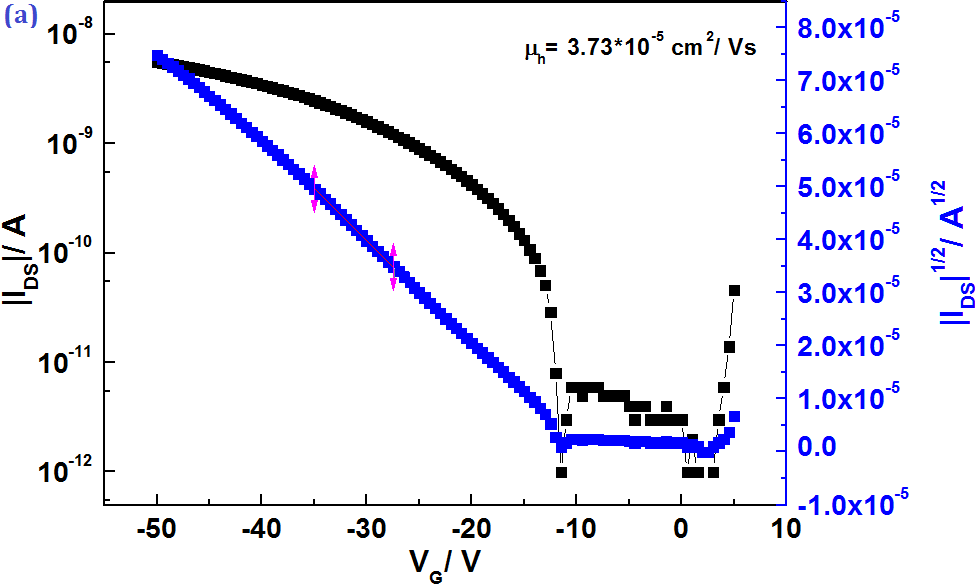
**

**
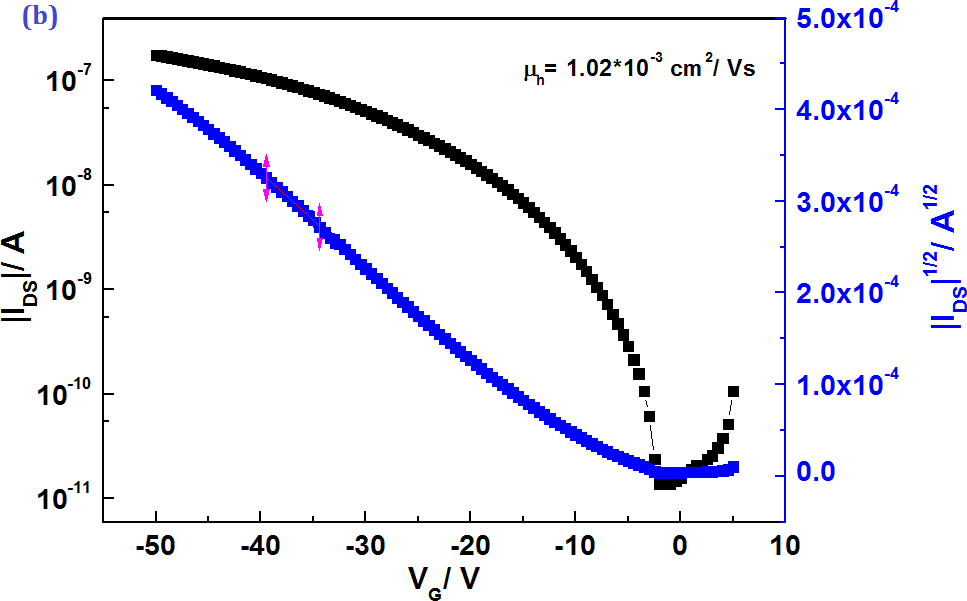
**


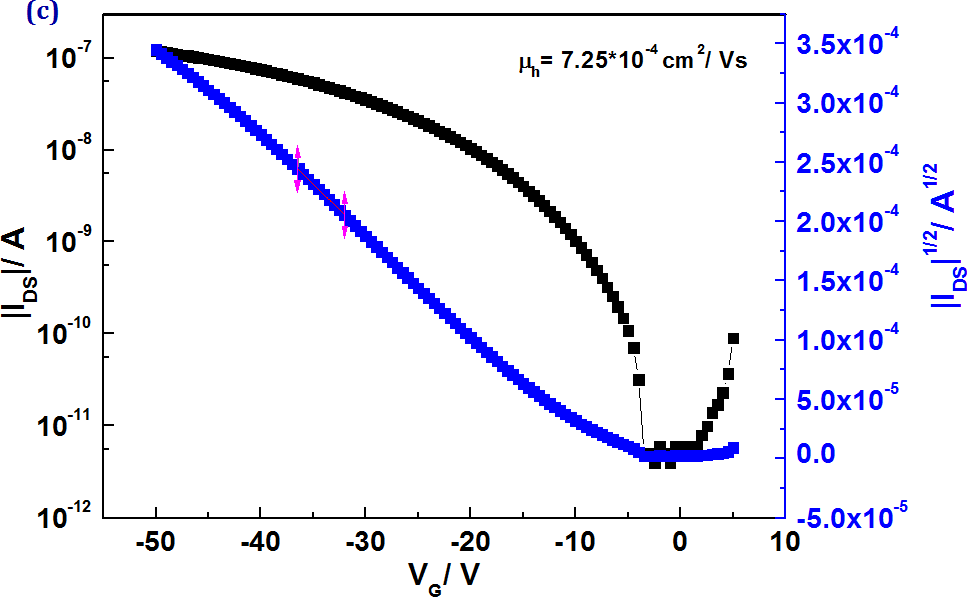


Figure S1. Transfer characteristics (measured at drain voltage of ‒50 V) of OFETs based on (a) PBDTC-TT1 and (b) PBDTC-TT2

**3. NMR spectra**

**
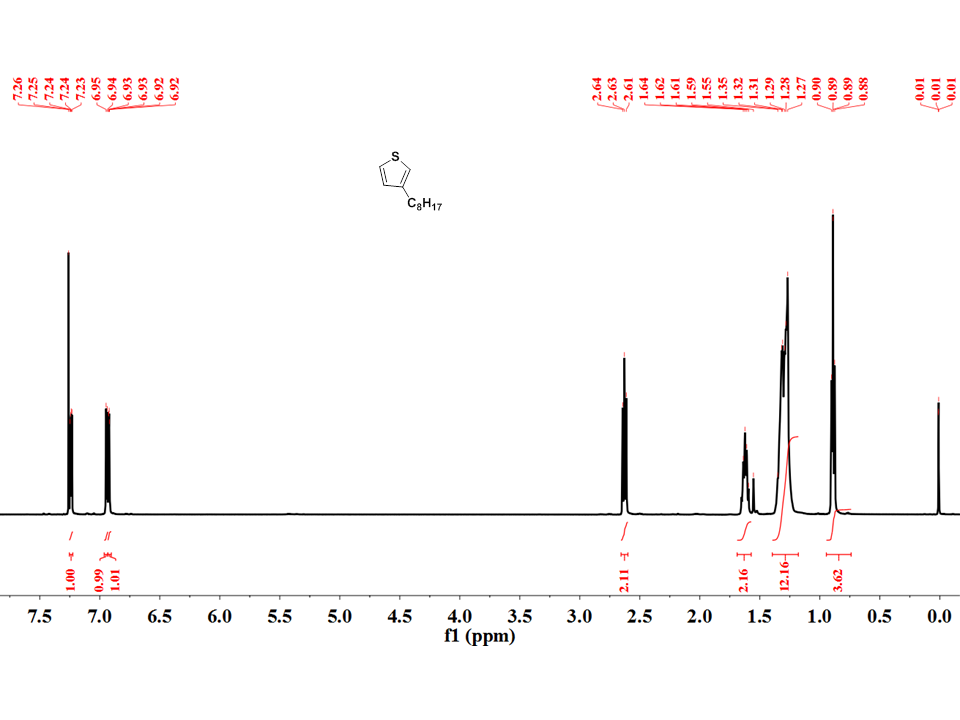
**

**Figure S2**. ^1^H NMR spectrum of **1.**


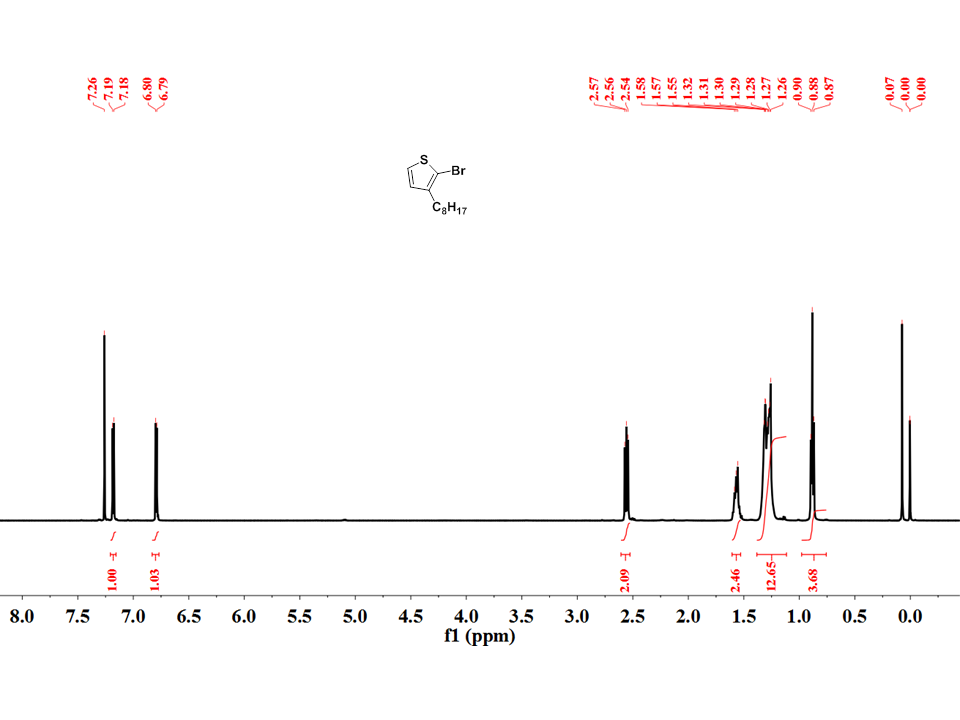


**Figure S3**. ^1^H NMR spectrum of **2.**


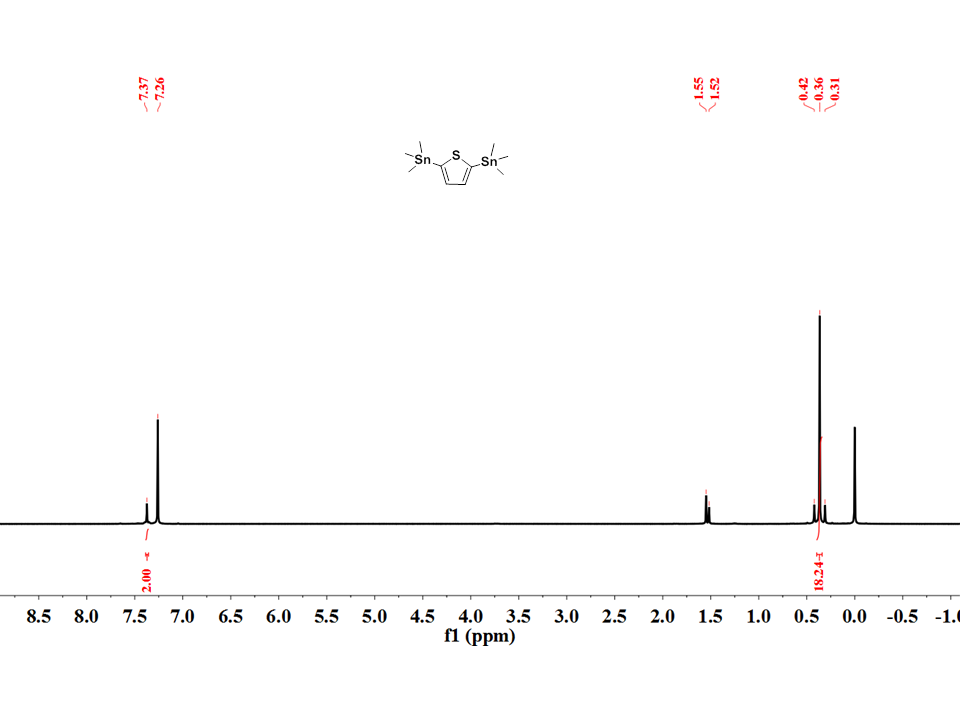


**Figure S4**. ^1^H NMR spectrum of **3.**


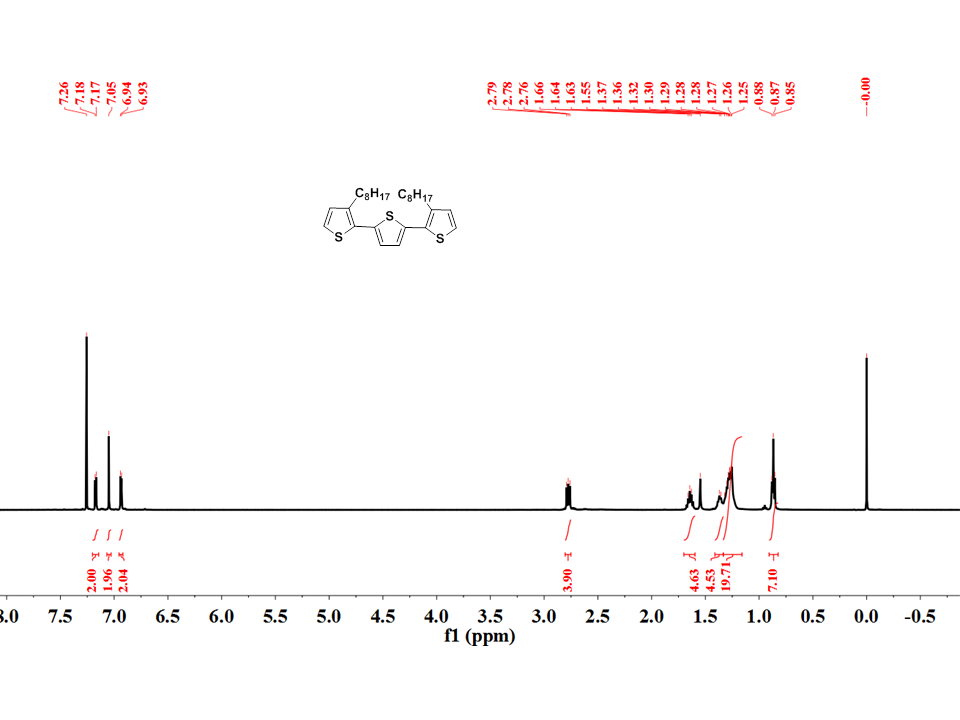


**Figure S5**. ^1^H NMR spectrum of **4.**


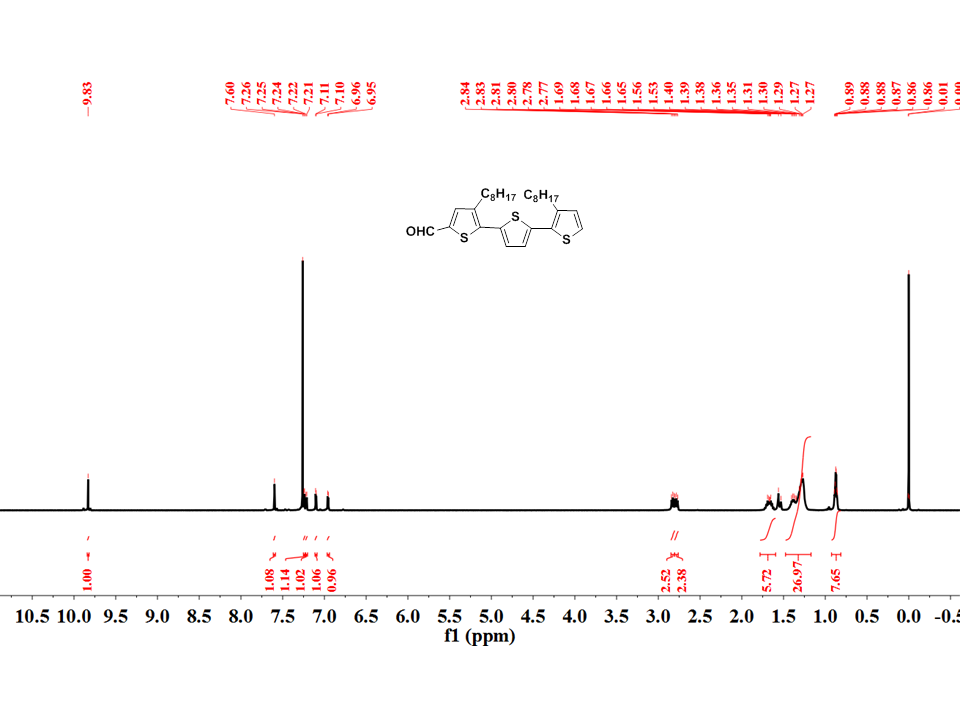


**Figure S6**. ^1^H NMR spectrum of **5.**


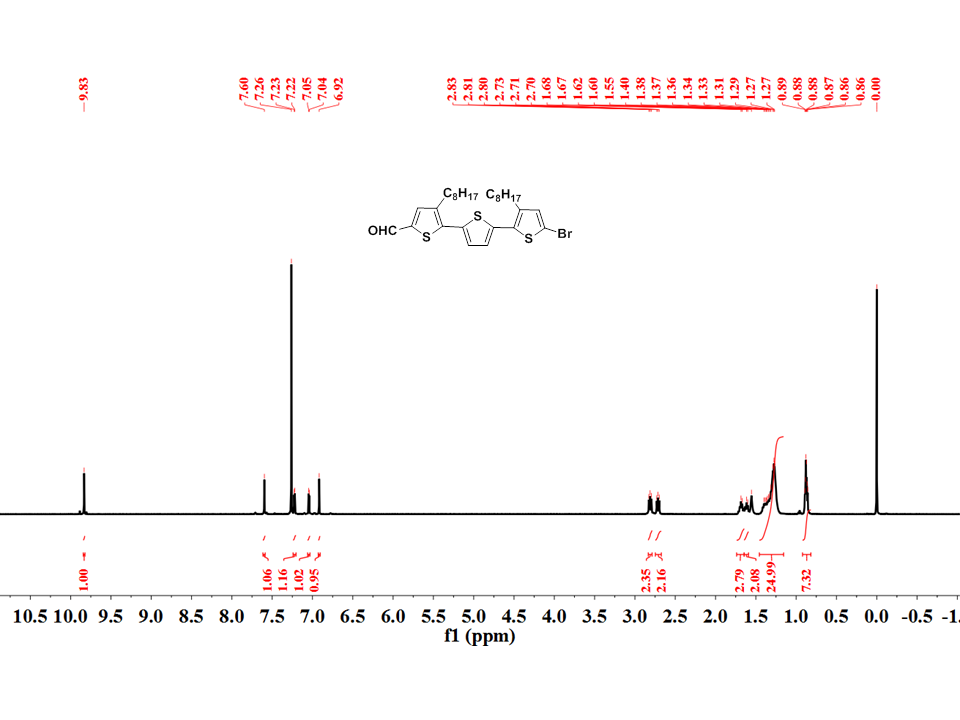


**Figure S7**. ^1^H NMR spectrum of **6.**

**
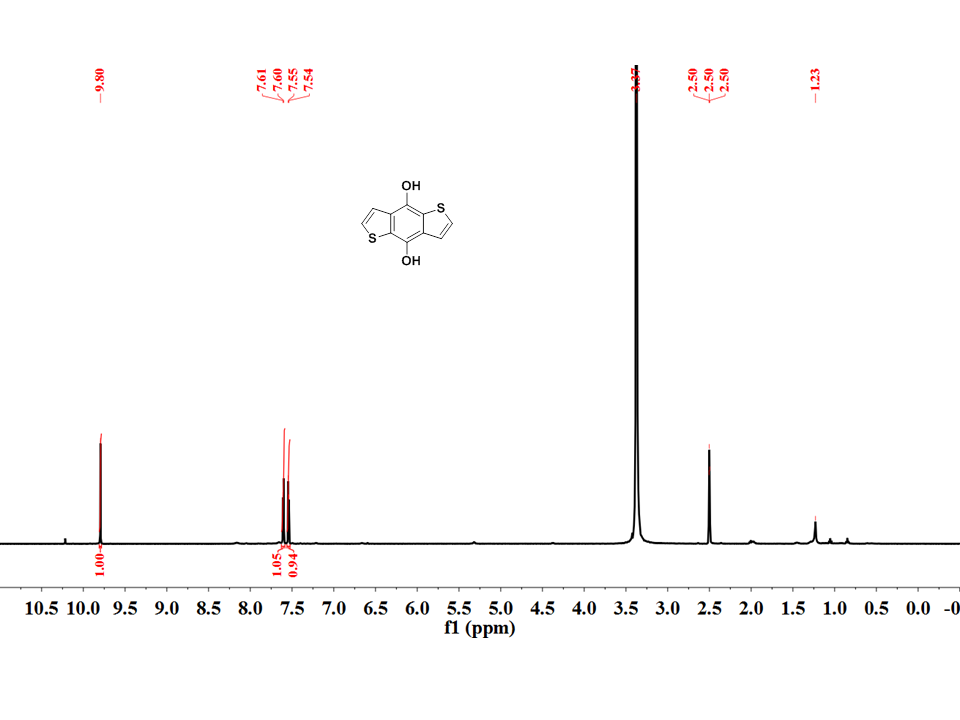
**

**Figure S8**. ^1^H NMR spectrum of **I**.


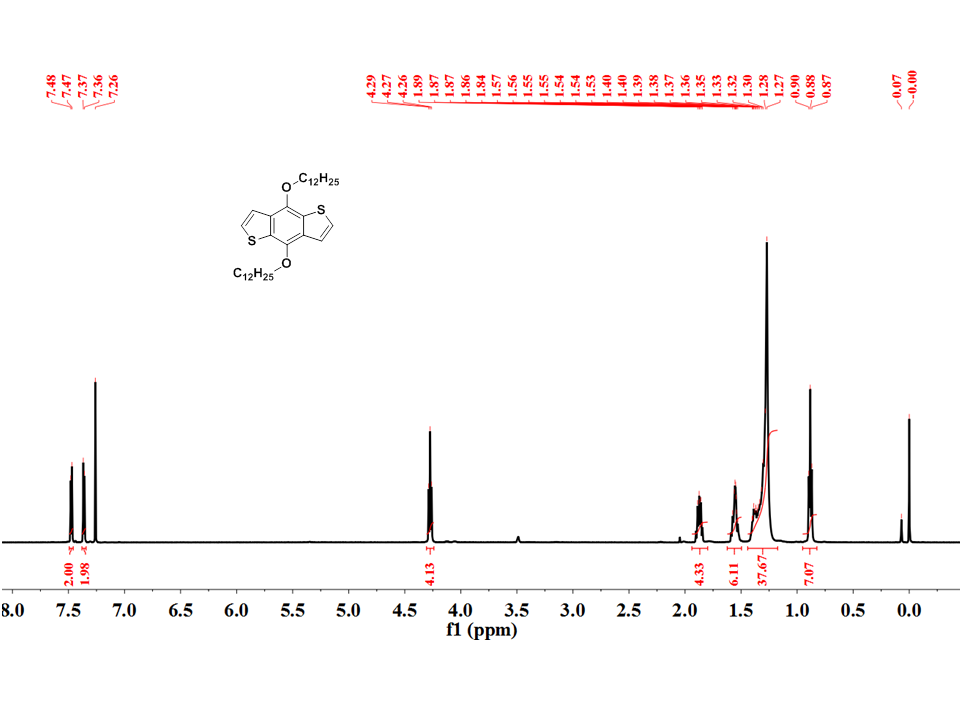


**Figure S9**. ^1^H NMR spectrum of **II.**


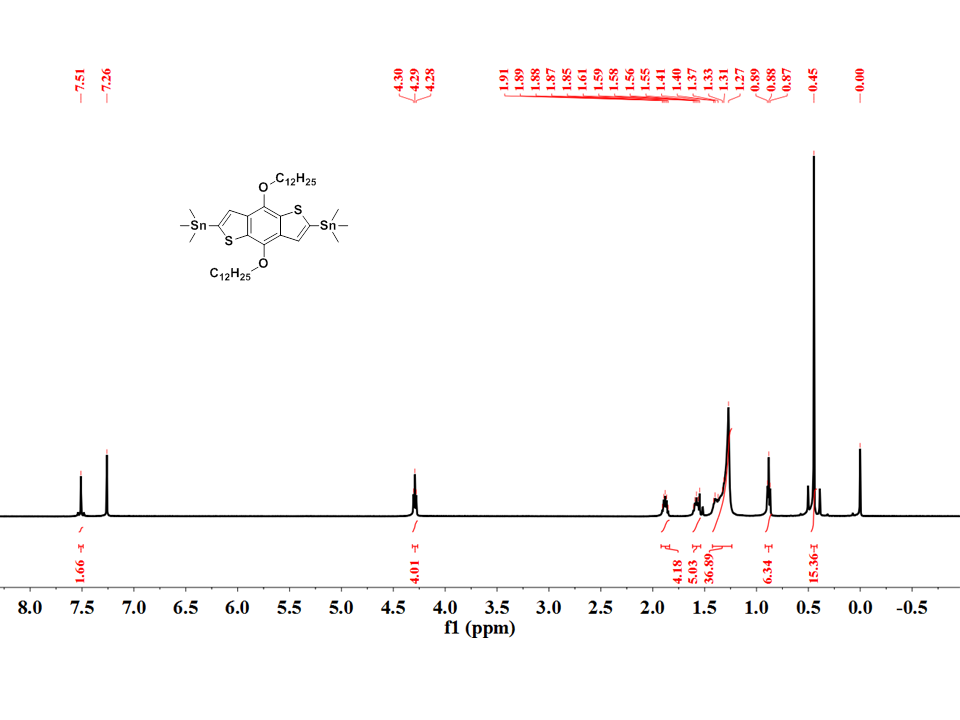


**Figure S10**. ^1^H NMR spectrum of **7.**


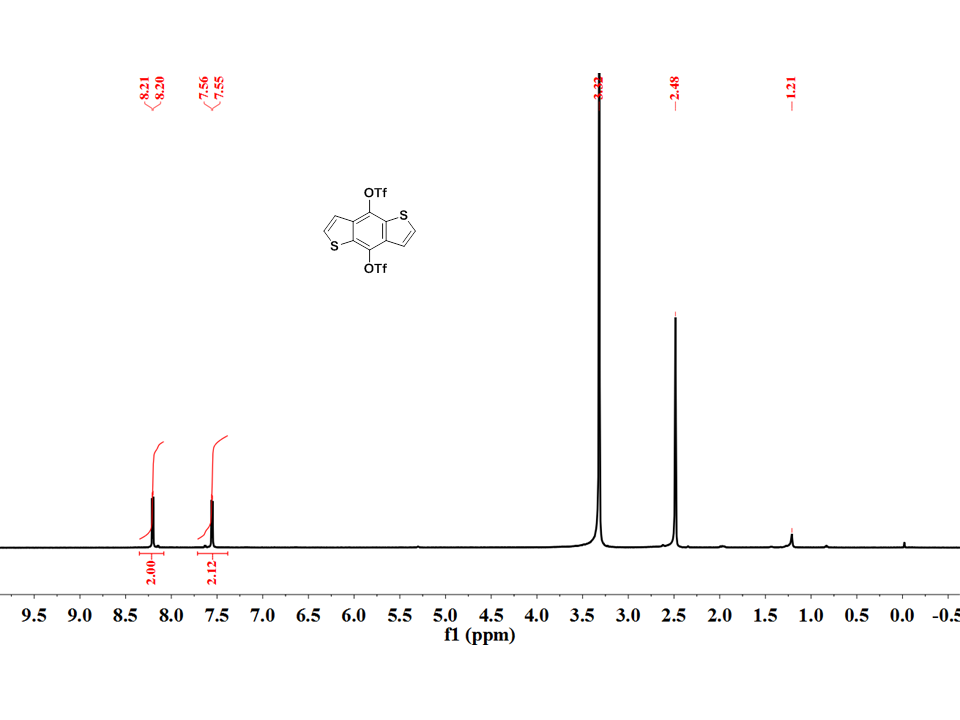


**Figure S11**. ^1^H NMR spectrum of **III.**


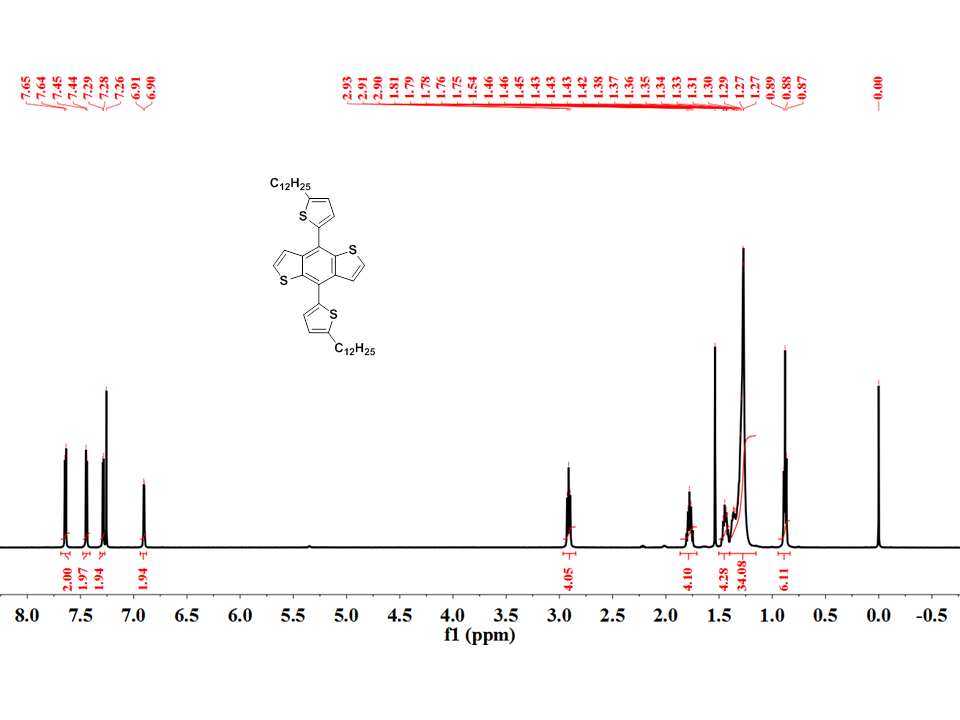


**Figure S12**. ^1^H NMR spectrum of **IV.**


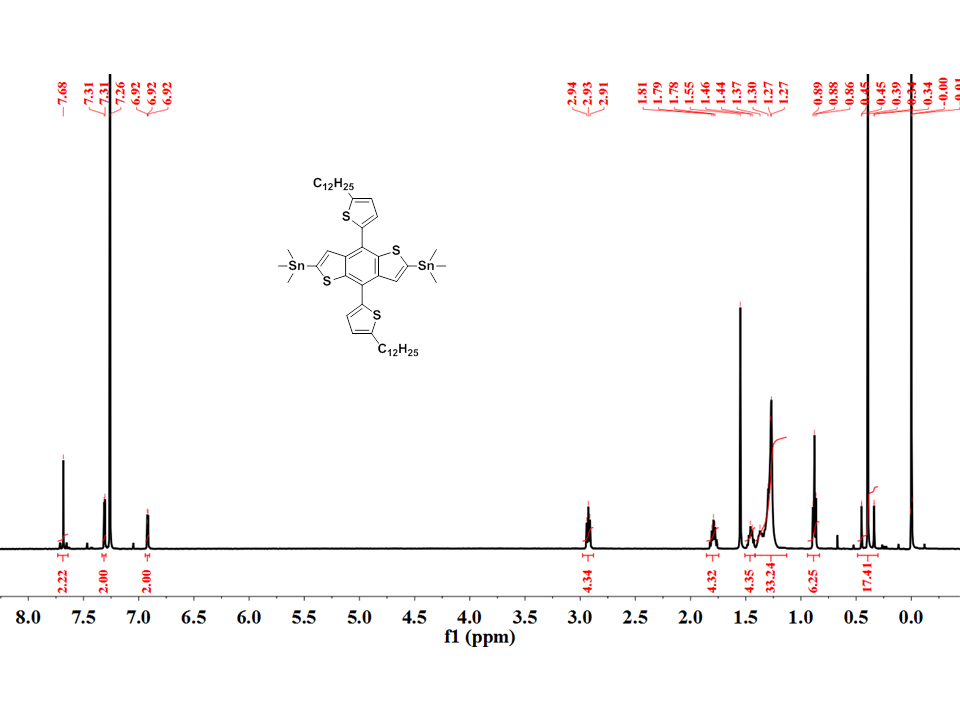


**Figure S13**. ^1^H NMR spectrum of **8.**


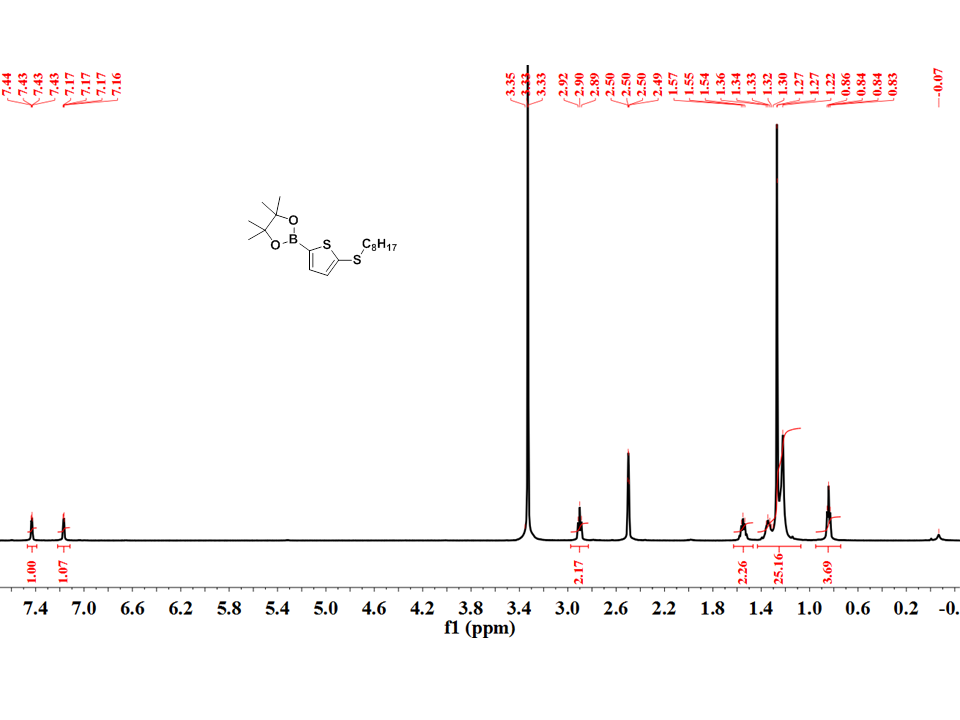


**Figure S14**. ^1^H NMR spectrum of **V.**


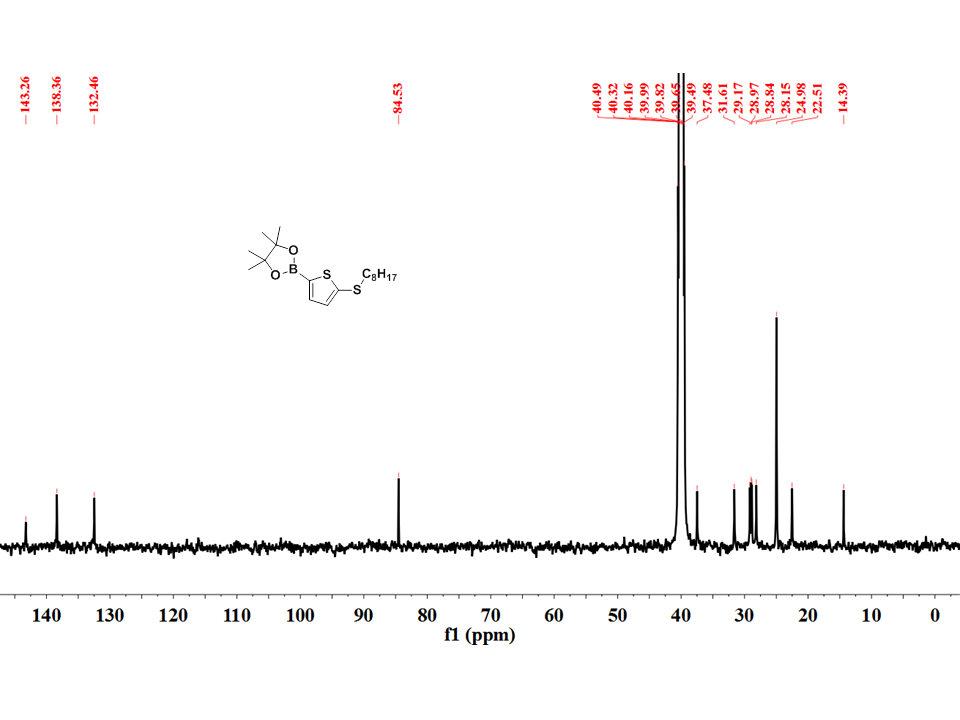


**Figure S15**. ^13^C NMR spectrum of **V.**


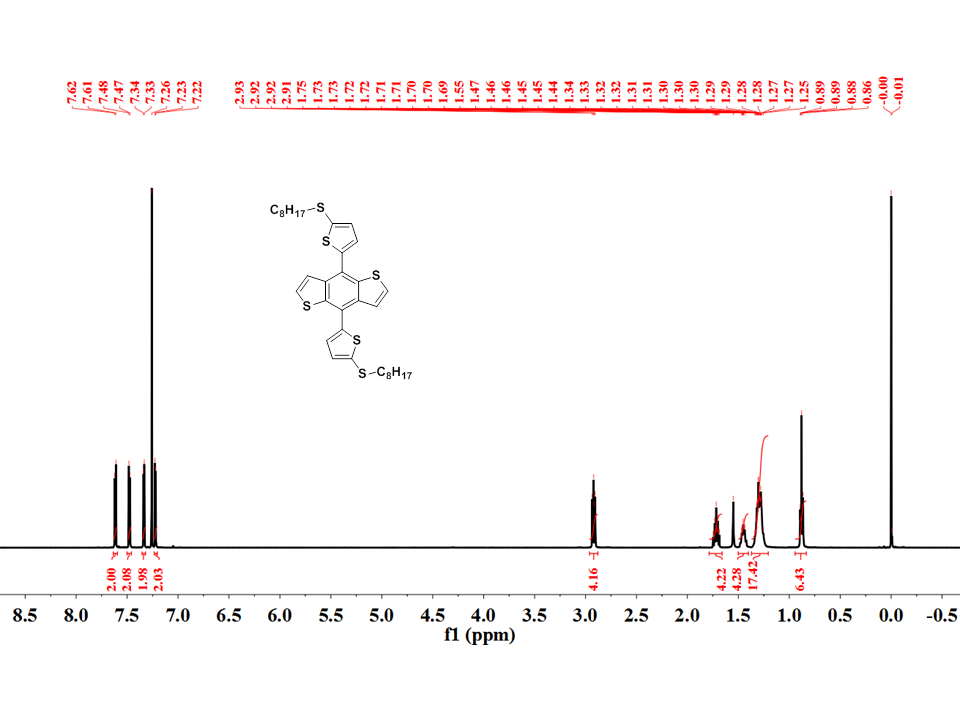


**Figure S16**. ^1^H NMR spectrum of **VI.**


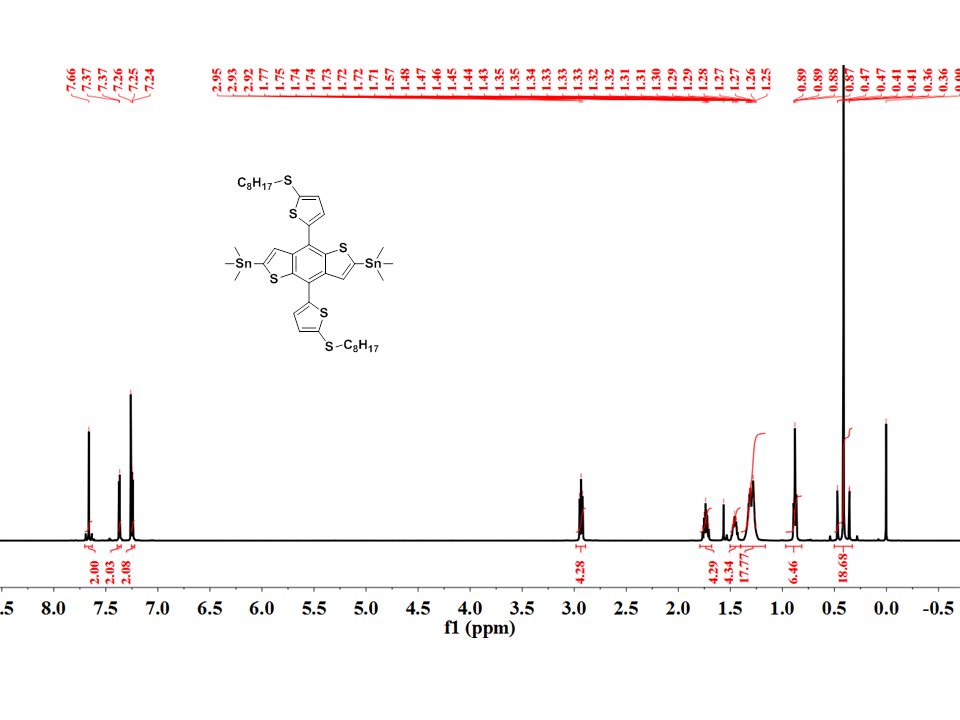


**Figure S17**. ^1^H NMR spectrum of **9.**


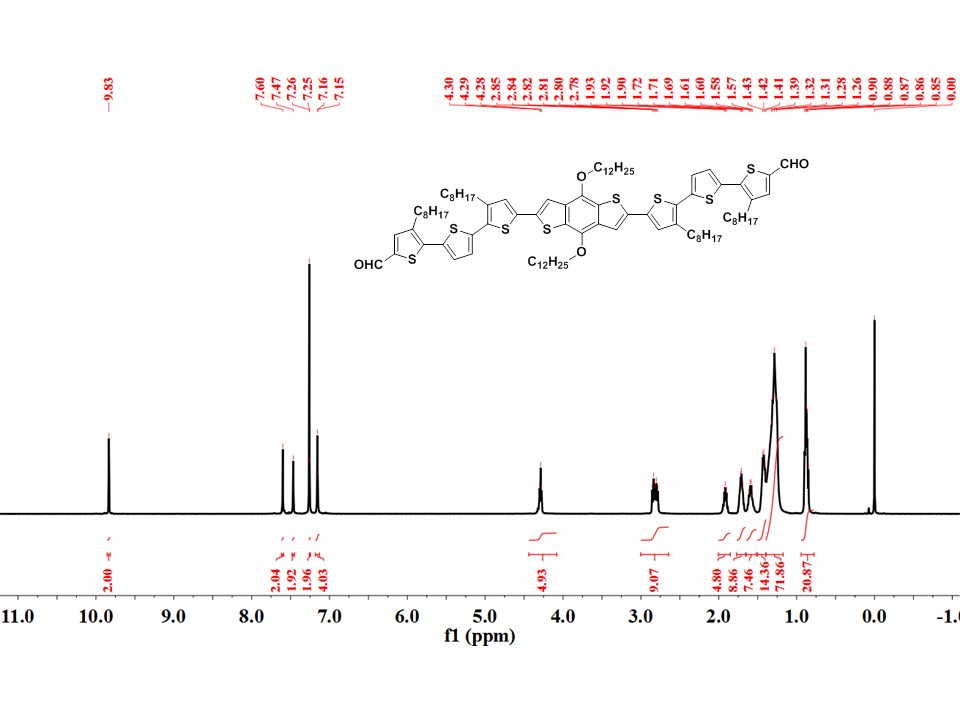


**Figure S18**. ^1^H NMR spectrum of **10.**


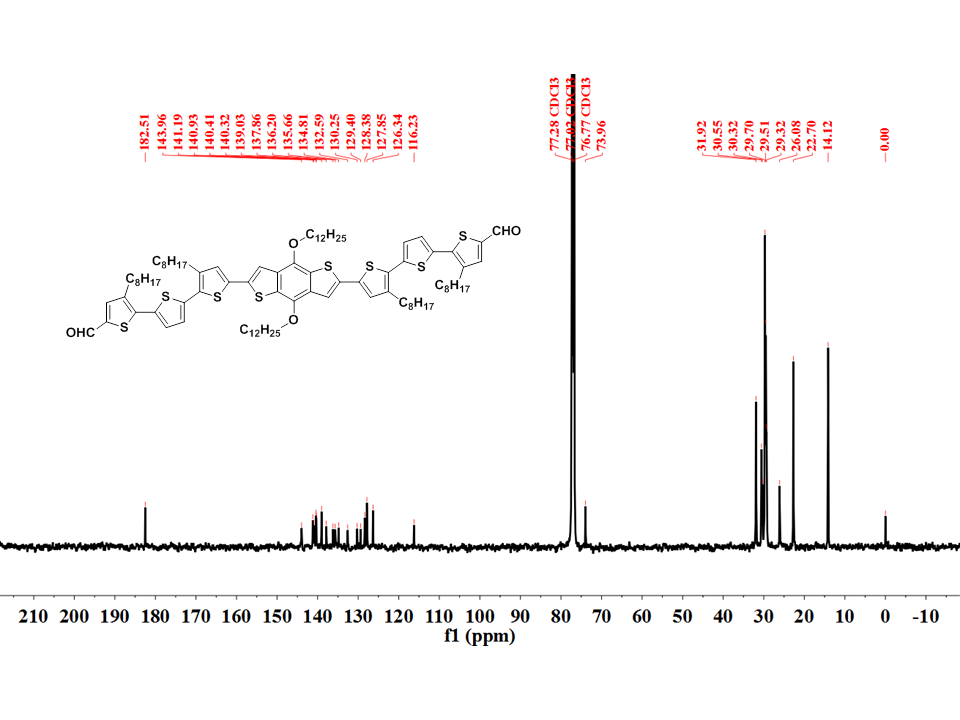


**Figure S19**. ^13^C NMR spectrum of **10.**


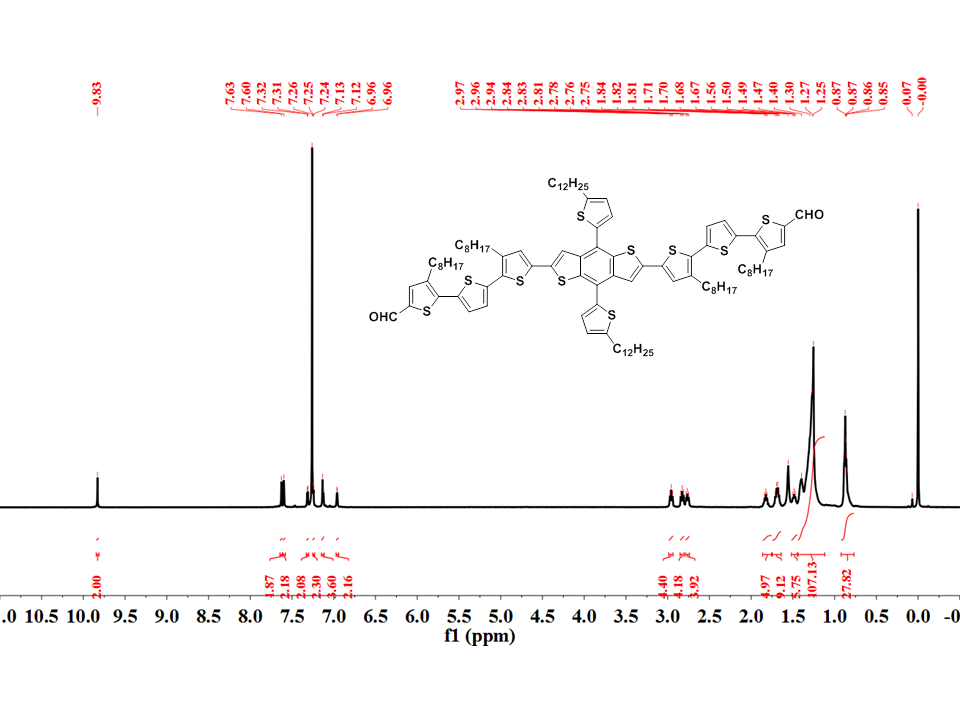


**Figure S20**. ^1^H NMR spectrum of **11.**


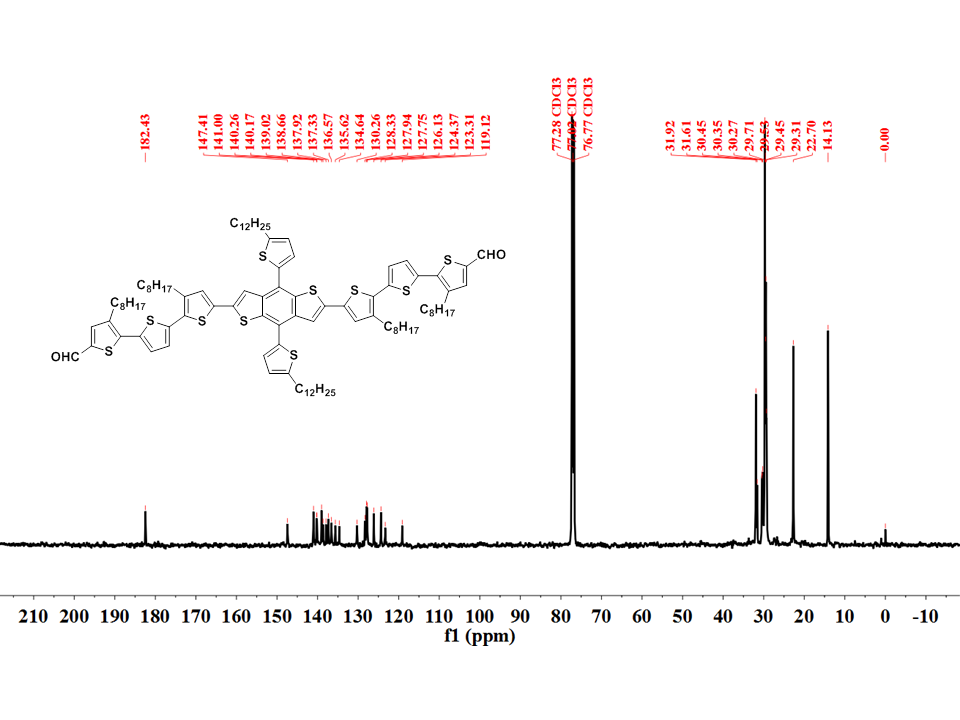


**Figure S21**. ^13^C NMR spectrum of **11.**


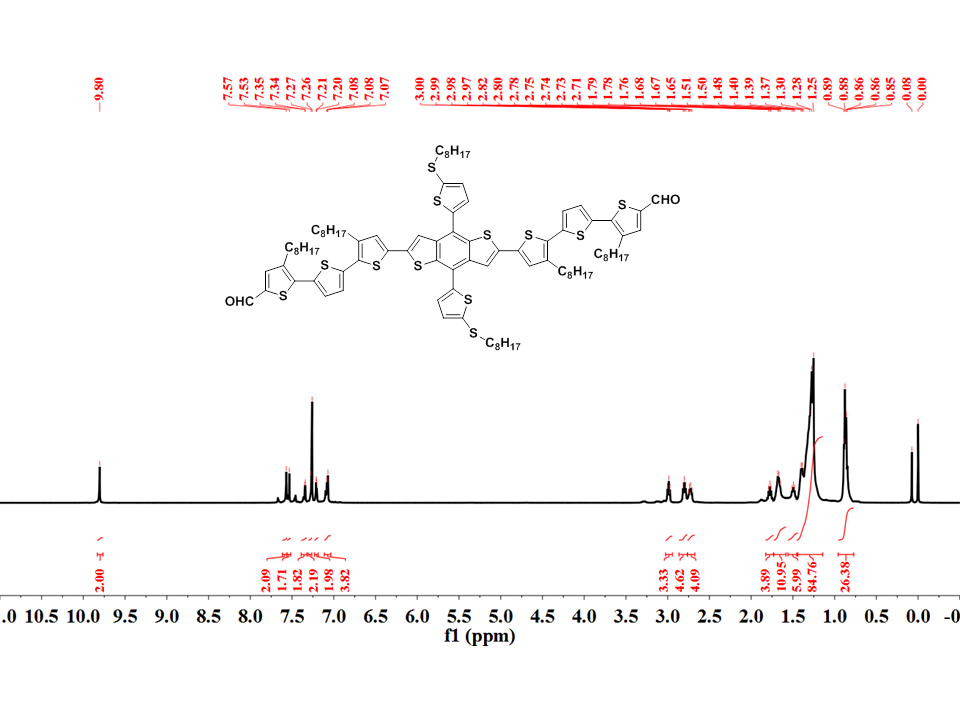


**Figure S22**. ^1^H NMR spectrum of **12.**


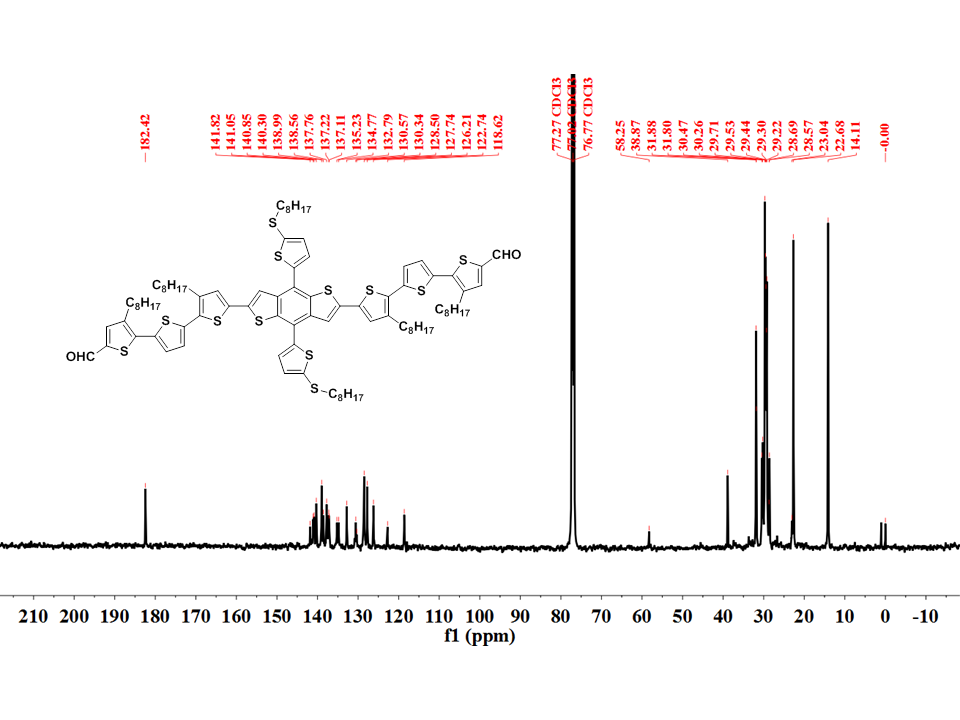


**Figure S23**. ^13^C NMR spectrum of **12.**


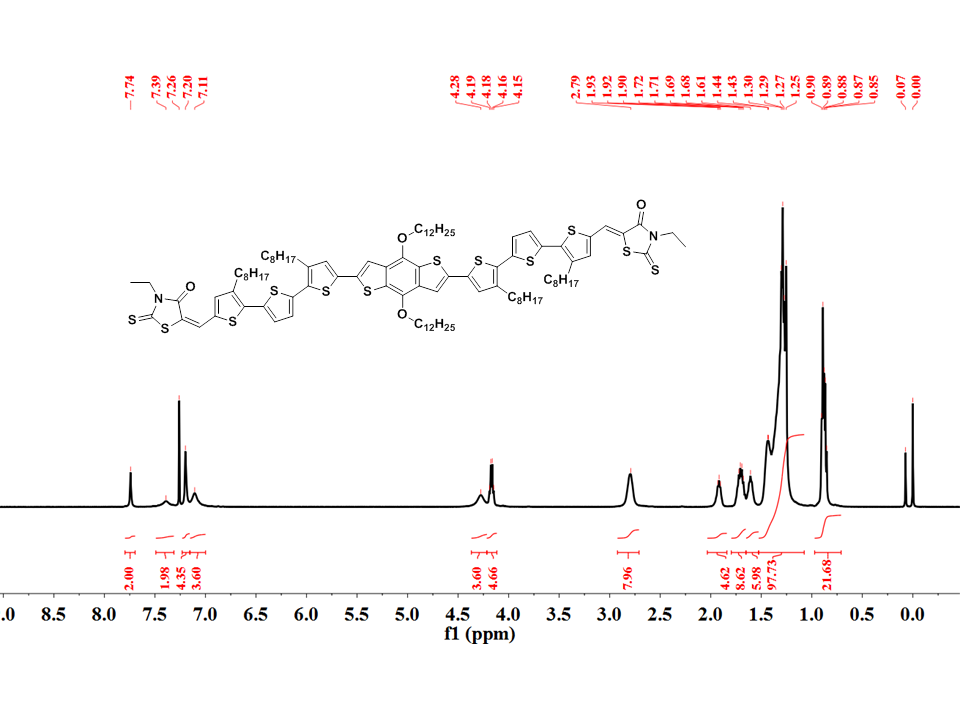


**Figure S24**. ^1^H NMR spectrum of **DR3TBDTOC12.**


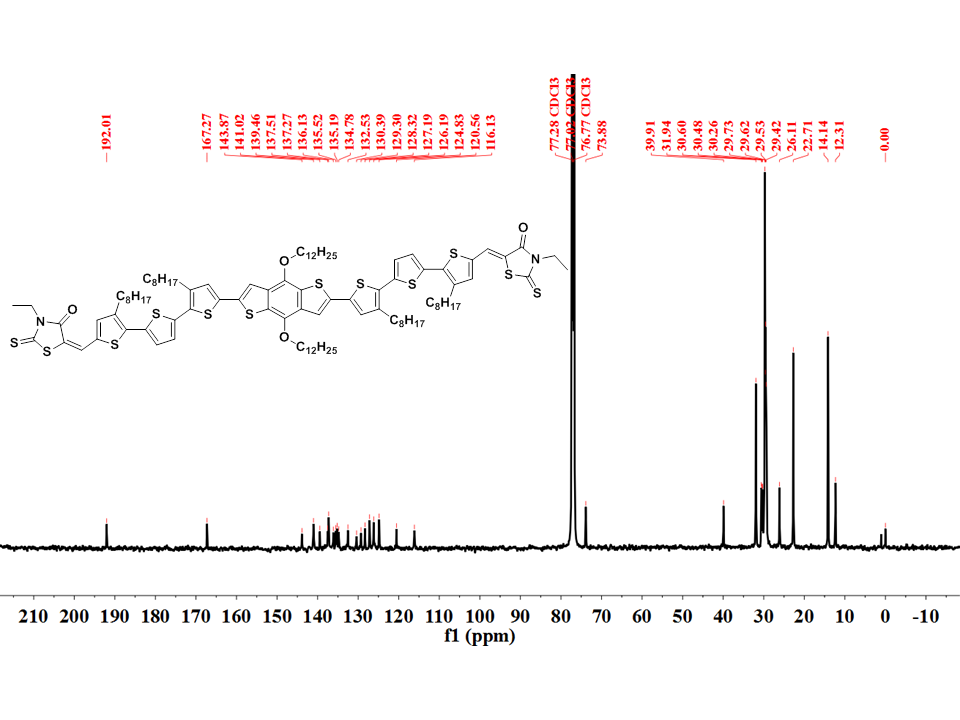


**Figure S25**. ^13^C NMR spectrum of **DR3TBDTOC12.**


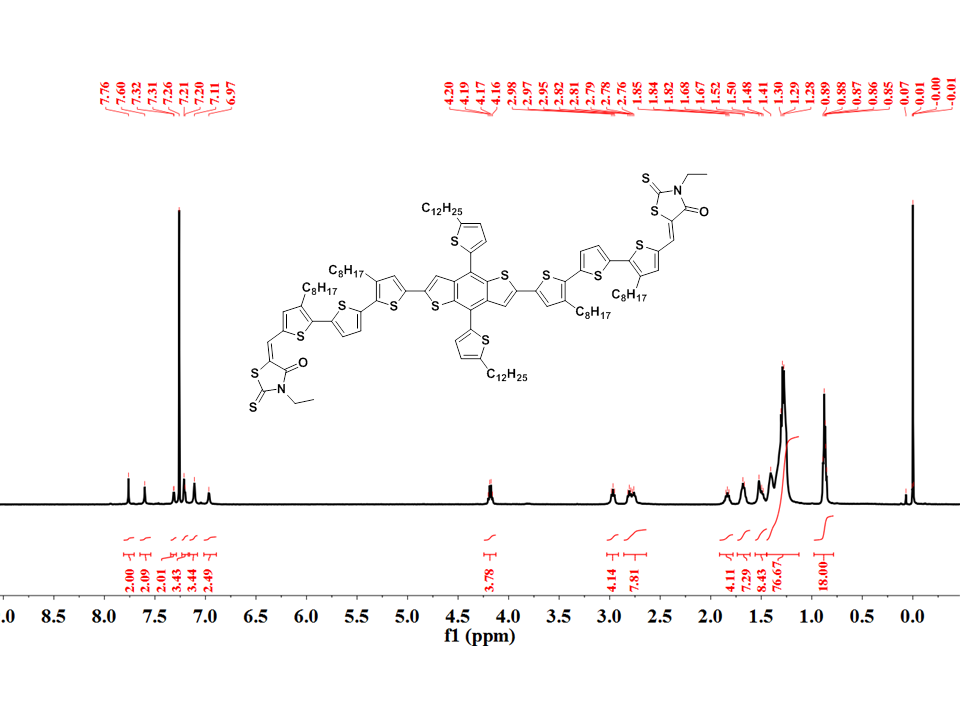


**Figure S26**. ^1^H NMR spectrum of **DR3TBDTTC12.**


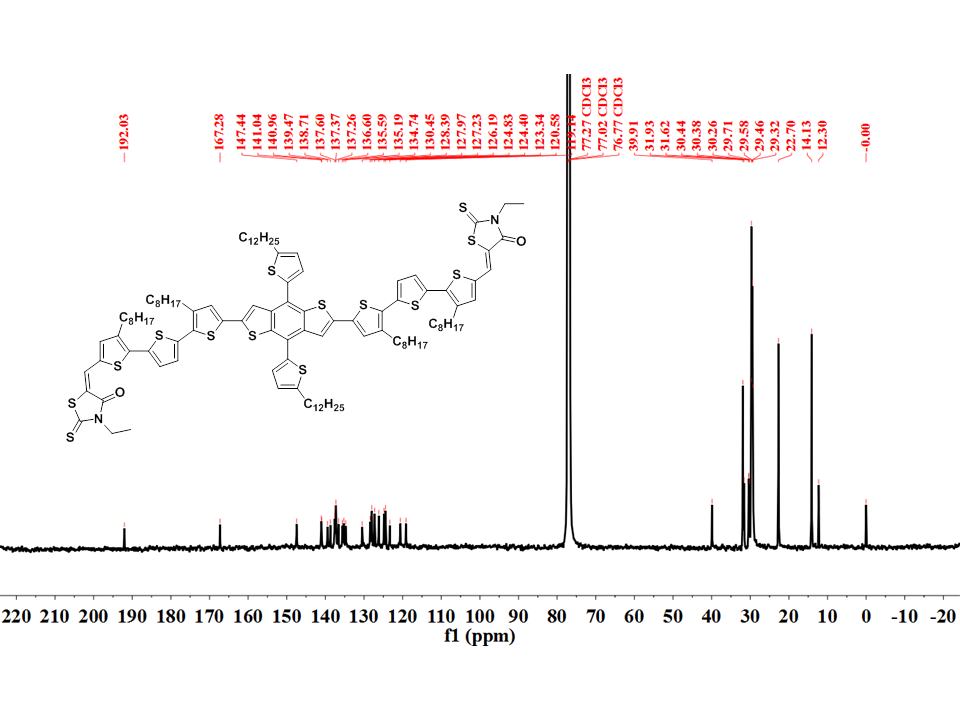


**Figure S27**. ^13^C NMR spectrum of **DR3TBDTTC12.**


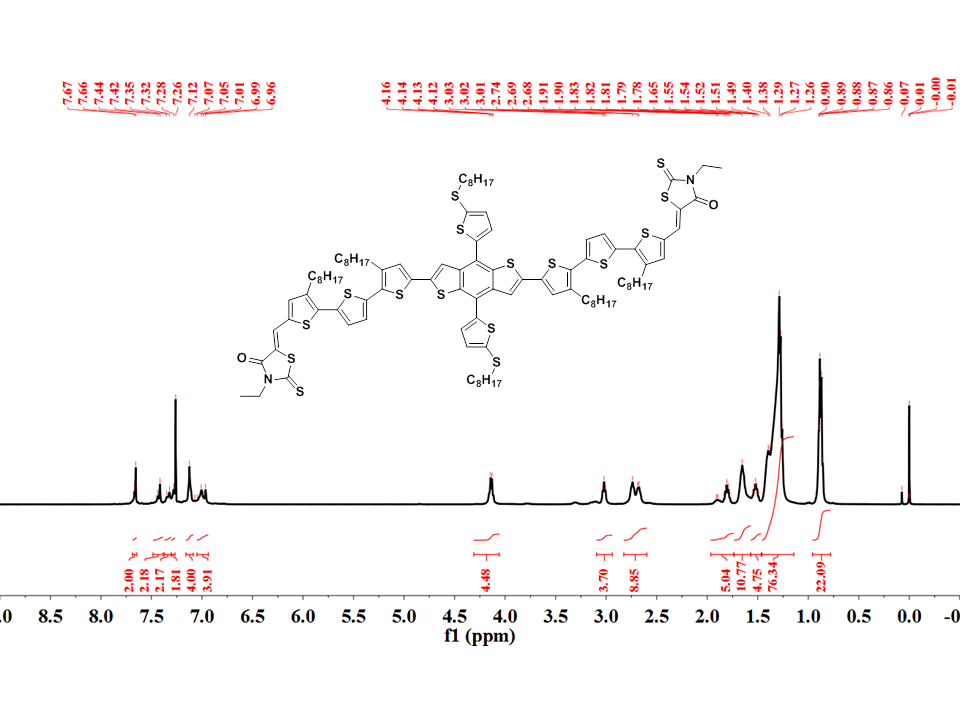


**Figure S28**. ^1^H NMR spectrum of **DR3TBDTTSC8.**


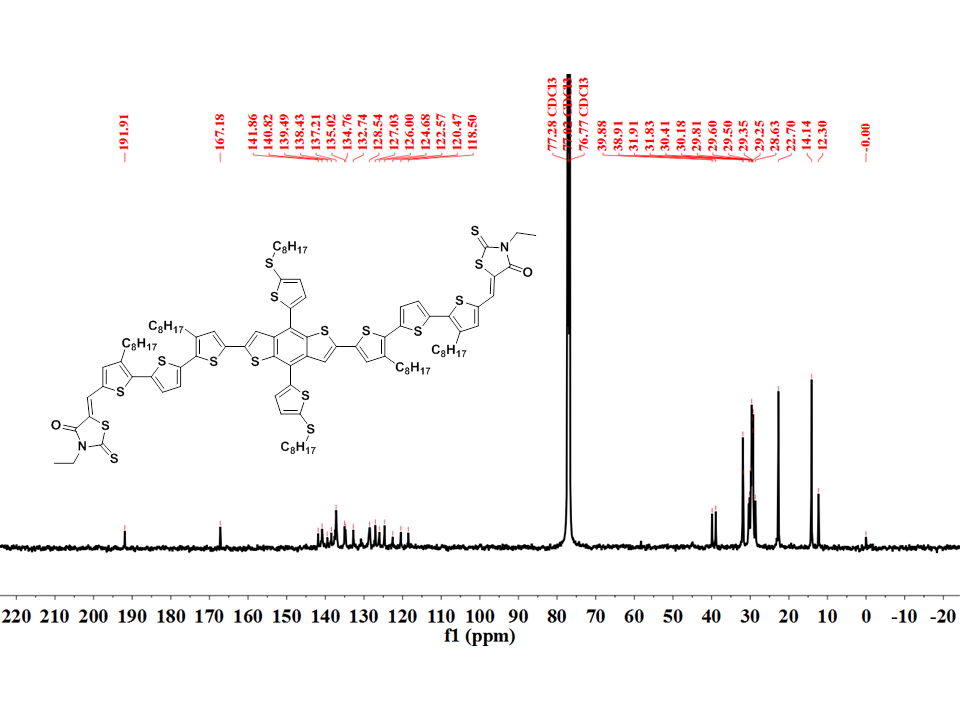


**Figure S29**. ^13^C NMR spectrum of **DR3TBDTTSC8**.
